# The Development and Evaluation of a Clinical Reasoning Case for Second-Year Medical Students

**DOI:** 10.15766/mep_2374-8265.11596

**Published:** 2026-04-28

**Authors:** Millie C. Kirchberg, Darcy Wooten, W. Cameron McGuire

**Affiliations:** 1 Internal Medicine Resident, Department of Medicine, University of California, Los Angeles School of Medicine; 2 Professor of Medicine, Department of Medicine, Washington University in St. Louis School of Medicine; 3 Assistant Professor of Medicine, Department of Medicine, University of North Carolina, Chapel Hill School of Medicine

**Keywords:** Clinical Reasoning/Diagnostic Reasoning, Gastroenterology, Case-Based Learning, Differential Diagnosis

## Abstract

**Introduction:**

Clinical reasoning is a cornerstone of diagnostic and therapeutic decision-making, yet it is infrequently taught in preclinical medical education and is often taught implicitly.

**Methods:**

To address this gap, we developed a clinical reasoning case focused on hematochezia as part of a year-long longitudinal clinical reasoning course for second-year preclinical medical students. The session combined large-group instruction with small-group learning activities that emphasized core reasoning skills, including problem representation, illness scripts, differential diagnoses, and diagnostic and management planning. A pre- and postintervention survey measured changes in students’ confidence and attitudes about clinical reasoning skills and concepts and knowledge of hematochezia.

**Results:**

Of the 123 medical students who attended the case, 92 completed both surveys (74.8% response rate). Confidence improved significantly in all domains (*P* < .01 for problem representation; *P* < .0001 for others). Students’ attitudes toward case-based learning also improved (*P* < .0001). Knowledge scores increased for all items, with 1 reaching statistical significance (*P* < .05).

**Discussion:**

Integrating formal clinical reasoning instruction into preclinical medical education through case-based learning is an effective strategy for teaching second-year medical students essential clinical reasoning skills.

## Educational Objectives

By the end of this case, students will be able to:

1. Construct a problem representation synthesizing key clinical data.
2. Develop a prioritized differential diagnosis for hematochezia.
3. Differentiate between upper and lower gastrointestinal bleeding using clinical clues.
4. Identify clinical features distinguishing Crohn's disease from ulcerative colitis.
5. Interpret laboratory, imaging, and endoscopic findings in suspected irritable bowel disease.
6. Propose an initial management and monitoring plan for a patient with Crohn's disease.

## Introduction

Clinical reasoning, the cognitive processes by which clinicians gather, synthesize, and apply patient data to make diagnostic and management decisions, is foundational to medical practice.^1,2^ Despite its centrality, clinical reasoning is often taught implicitly in undergraduate medical education, particularly during the preclinical years.^3^ Traditional preclinical curricula tend to prioritize biomedical knowledge acquisition over instruction in higher-order reasoning processes, leaving many students underprepared for the analytic demands of clinical clerkships.^3–6^ Early, explicit instruction in clinical reasoning offers an important opportunity to foster deeper understanding of patient care, strengthen diagnostic thinking, and allow learners to practice decision-making in a structured, low-risk environment.

Several complementary learning theories help explain how clinical reasoning develops and how it can be taught effectively. Dual process theory conceptualizes reasoning as movement between rapid, intuitive pattern recognition (Type 1 thinking) and slower, more deliberate analytic processes (Type 2 thinking).^7^ Script theory describes how learners organize biomedical knowledge into illness scripts (structured mental representations linking epidemiology, pathophysiology, and clinical features) that are activated, refined, and compared when generating differential diagnoses.^8^ For novice learners, explicit instruction in problem representation and illness script development may support more systematic diagnostic thinking. Cognitive apprenticeship further provides a framework for teaching clinical reasoning in authentic contexts by making expert thinking visible through modeling, coaching, scaffolding, articulation, reflection, and gradual transfer of responsibility.^5^ Finally, Kolb's experiential learning model supports teaching of clinical reasoning by guiding learners through iterative cycles, making the cognitive steps of diagnostic thinking explicit, practiced, and progressively refined.^9^ These theories emphasize that clinical reasoning is both developmental and learnable when expert processes are intentionally externalized.

Although prior interventions have used case-based learning and facilitated small-group discussion to teach clinical reasoning to preclinical students, relatively few explicitly describe how educational theory informs the design of individual cases.^10^ Even fewer deliberately integrate multiple complementary theories within a single instructional intervention.^11,12^ This gap limits educators’ ability to intentionally align learning objectives, instructional strategies, and assessment with the cognitive processes underlying clinical reasoning.

In response, we developed a longitudinal clinical reasoning course for second-year medical students embedded within an organ-system–based preclinical curriculum. The course integrates principles from dual process theory, script theory, cognitive apprenticeship, and Kolb's experiential learning cycle. Each weekly session centers on 1 or 2 high-fidelity clinical cases delivered in sequential “aliquots,” allowing learners to iteratively generate hypotheses, refine problem representations, revise differential diagnoses, and determine appropriate diagnostic or management steps as new data emerge. Working in small groups, students engage in structured reasoning tasks, followed by facilitated debriefs in which faculty explicitly model expert clinical reasoning before advancing the case. Alignment of cases with concurrent organ-system content provides repeated opportunities for deliberate practice, reflection, and progressive refinement of reasoning skills across 2 academic quarters.

Here, we describe 1 representative case from this course: a hematochezia-focused case centered on the diagnosis and inpatient management of Crohn's disease. This exploratory pilot study focused on feasibility and student perceptions of the case. Hematochezia was selected because it is a common and clinically meaningful presentation that requires integration of epidemiology, pathophysiology, risk stratification, and comparison of overlapping illness scripts, including inflammatory bowel disease, infectious colitis, diverticular bleeding, and upper gastrointestinal bleeding with rapid transit. This case illustrates how multiple educational theories can be intentionally operationalized within a single instructional design to support the development of clinical reasoning in preclinical medical students.

## Methods

### Curricular Context

We embedded a hematochezia case within a newly developed longitudinal clinical reasoning course for second-year medical students enrolled in a block-based, organ-system preclinical curriculum. We designed the course using the Kern Model for Curriculum Development^13,14^ and sought to (1) introduce a standardized framework for core clinical reasoning processes; (2) provide frequent opportunities for deliberate practice in a low-stakes environment; (3) deliver timely formative feedback using an assessment-for-learning approach; (4) simulate authentic clinical encounters to promote transfer of skills; and (5) ensure diversity across patient populations, disease processes, and clinical settings.

The instructional design integrated multiple complementary theories of learning and reasoning. Drawing on dual process theory, we structured cases to prompt early hypothesis generation from limited clinical data, followed by deliberate analytic reasoning as additional information was sequentially revealed. Script theory informed case construction by emphasizing the generation, comparison, and refinement of illness scripts through prioritized differentials and identification of supporting and refuting clinical data. Cognitive apprenticeship guided instructional delivery, with faculty explicitly modeling expert diagnostic and management reasoning during structured debriefs, while scaffolding learner participation through guided prompts and small-group tasks. Kolb's experiential learning cycle was incorporated through repeated cycles of concrete experience (high-fidelity cases), reflection (small-group discussion and debrief), abstraction (explicit reasoning frameworks), and active experimentation across subsequent cases.

### Case Development

Within the course, we developed weekly cases using predefined criteria to ensure breadth, authenticity, and educational value. Specifically, cases were chosen to represent common clinical syndromes (eg, chest pain, dyspnea, abdominal pain, fever), span the age spectrum, include both medically and surgically oriented disease processes, and reflect conditions evaluated across ambulatory, inpatient, and intensive care settings. Presenting chief concerns and case narratives were deliberately written to mirror real-world clinical practice and generate broad differential diagnoses. We planned case details and embedded nuance in a progressive fashion to prompt learners to compare and contrast competing diagnostic hypotheses and iteratively refine their reasoning as new data emerged. Although the primary emphasis was on diagnostic reasoning, cases also incorporated introductory management reasoning tasks appropriate for early learners. These included prompts related to laboratory and imaging selection, attention to pretest probability and high-value care, and empiric management decisions. Weekly cases were thematically aligned with organ-system content taught 1–2 weeks prior, reinforcing integration with the concurrent curriculum. The hematochezia case (Appendix A), centered around the diagnosis and inpatient management of a patient ultimately diagnosed with Crohn's disease, was delivered during the Gastroenterology organ-system block.

### Instructional Delivery

We delivered the case in a team-based, large-group format interspersed with facilitated small-group activities. Students participated in a brief large-group didactic session (Appendix B), followed by small-group breakout exercises (Appendix C) focused on constructing problem representations, generating and refining differential diagnoses, identifying supporting and refuting data, and proposing diagnostic and management plans. Following each activity, we debriefed the group by explicitly articulating their clinical reasoning before progressing to subsequent case aliquots, consistent with a cognitive apprenticeship approach. The session spanned 2.5 hours, although this time was not specified a priori but rather resulted from the natural evolution of the case discussion and instruction. This case was delivered partway through the 2-quarter longitudinal course and approximately 3 months into the students’ second year. It was the ninth of 31 cases covered during the course, so students had reasonable experience with session logistics.

All instructional materials, including patient narratives, lecture slides, small-group activities, and assessment instruments, were developed by a fourth-year medical student (Millie Kirchberg) as an independent scholarly medical education project. Internal Medicine faculty (Darcy Wooten and W. Cameron McGuire) provided supervision and iterative feedback throughout development, and final materials were reviewed and approved by Millie Kirchberg's interdisciplinary scholarly oversight committee.

### Participants

Participants included second-year medical students enrolled at the University of California, San Diego School of Medicine during the 2024–25 academic year. All students had brief exposures to patient care activities through their monthly half-day sessions with longitudinal preceptors throughout the first year of medical school.

### Surveys

Pre- and postintervention surveys (Appendices D and E, respectively) were administered via Qualtrics (October 2024 version; Provo, UT) to assess changes in students’ confidence, attitudes, and knowledge regarding clinical reasoning and the evaluation of hematochezia. Survey items were developed in accordance with best-practice guidelines for medical education survey design outlined by Artino and colleagues,^15^ with attention to clarity, construct alignment, and response process validity. Confidence was assessed using a 7-point Likert scale to capture nuanced changes in self-assessment, whereas attitudes were measured using a 5-point Likert scale. Knowledge was evaluated using multiple-choice questions focused on diagnostic and inpatient management decisions relevant to the case. We asked a single qualitative question at the end of the survey asking what could be done to improve the case for future sessions. Survey participation was voluntary, anonymous, and without incentive. The University of California, San Diego Institutional Review Board determined that the study was exempt (IRB #812812). The first author (Millie Kirchberg), who had no evaluative role in the course and no personal relationship with the second-year students, managed all survey data. She matched the pre- and postsurveys using student names and removed all personal identifiers before presenting the data to the senior author (W Cameron McGuire) for statistical analysis to ensure anonymity for the students.

### Statistical Analysis

Paired Student's *t* tests were used to evaluate pre- and postintervention differences in confidence and attitude measures derived from Likert-scale items. Knowledge-based items were dichotomized as correct or incorrect and analyzed using McNemar's test to assess within-subject changes in performance. Descriptive and inferential analyses for Likert-scale data were performed using Microsoft Excel (Version 16.94; Microsoft Corporation, Redmond, WA). McNemar's tests were conducted using SPSS (Version 29.0.2.0; IBM Corporation, Armonk, NY). A 2-sided *P* value < .05 was considered statistically significant.

## Results

Of the 142 second-year medical students, 123 participated in the hematochezia clinical reasoning session. Of these, 92 students completed both the pre- and postintervention survey (74.8% response rate). The pre- and postsurvey questions centered around Kirkpatrick's evaluation framework.^16^ Confidence and attitudes were treated as components of Level 1: Reaction, reflecting learner perceptions and self-reported confidence with applying clinical reasoning skills. Knowledge-based questions corresponded to Level 2: Learning, assessing changes in applied understanding of hematochezia evaluation and management.

Students’ confidence in their clinical reasoning skills (developing problem representations, differential diagnoses, illness scripts, laboratory and imaging workup, interpreting diagnostic results, and creating a treatment plan), improved significantly after the session (Table 1, *P* < .01 for creating a problem representation and *P* < .0001 for all other domains).

**Table 1.**
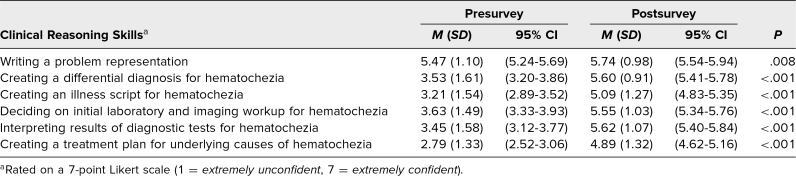
Mean Confidence in Clinical Reasoning Skills Between Pre- and Postsurveys (*N* = 92)

Students’ perceptions of the value of case-based learning exercises in enhancing their clinical reasoning skills for hematochezia significantly increased (*P* < .0001). There was a trend towards a significant increase in the belief that (1) clinical reasoning cases prepare students for clerkships and (2) small-group learning is preferred over individual learning (*P* < .051 and *P* < .052, respectively).

In addition, medical students’ knowledge improved throughout the case. The correct response rates for 3 knowledge-based questions were 78%, 88%, and 93% on the presurvey and 88% (*P* < .012), 91% (*P* < .791), and 100% (*P* < .063), respectively, on the postintervention survey.

Students reported high satisfaction with their learning experiences from this case, and all postintervention survey respondents felt that the case was appropriate for their level of training. Most students agreed or strongly agreed that the session helped improve their clinical reasoning skills and ability to distinguish ulcerative colitis from Crohn's disease. They also reported an increase in their confidence with developing a management plan for Crohn's disease and felt that the session helped prepare them for third-year clerkship rotations (Table 2). Twenty students (21.7%) provided a free-text comment for the qualitative question asking what could be done to improve the case for future sessions. Two major themes emerged from these answers. Seven respondents wanted the small-group discussion times shortened, and 3 respondents wanted more faculty facilitators for the small-group portions of the case.

**Table 2.**
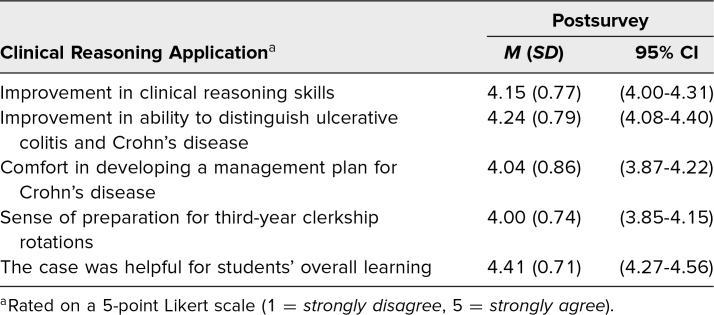
Mean Attitudes About Effectiveness of Various Case Metrics (*N* = 92)

## Discussion

Our clinical reasoning case effectively enhanced medical students’ confidence, attitudes, and knowledge related to clinical reasoning and the management of hematochezia and Crohn's disease. The improvements in students’ confidence across all domains suggest that structured, case-based learning interventions can positively influence students’ ability to engage in key components of clinical reasoning. Additionally, the increased appreciation for case-based learning underscores the importance of interactive and applied educational methodologies in medical training.

These findings align with prior research highlighting the role of structured, case-based teaching in fostering clinical reasoning skills among medical trainees. Several studies have shown that clinical reasoning instruction can improve diagnostic accuracy, enhance problem-solving abilities, and better prepare students for the challenges of clinical rotations.^17–19^ Similarly, we noticed a statistically significant improvement in students’ confidence, leading to a prioritized differential diagnosis and overall improvements in their confidence, attitudes toward clinical reasoning, and knowledge related to the evaluation and management of hematochezia. While confidence and comfort do not equal competence or comprehension, these results, when interpreted in the context of additional literature on the topic, support the inclusion of clinical reasoning instruction during preclinical training as a strategy to build foundational skills before students enter clerkships. Future research could explore whether exposure to a hematochezia case during the second year improves students’ ability to conduct an appropriate workup and management of hematochezia either in a simulated case or clinical practice during the third year.

Many medical education studies emphasize subjective outcomes like learner confidence and attitudes.^20–23^ When knowledge is assessed, it is typically through standardized testing following didactic instruction. In contrast, our case-based session did not employ traditional lecture formats or explicitly teach the material that was later assessed. Instead, it evaluated students’ knowledge gained through engagement with the case itself. While correct answers for the knowledge-based questions were high in the presurvey, the percentage of correct answers increased in the postsurvey, suggesting at least some degree of knowledge acquisition.

We believe that the combination of lecture-based instruction interspersed with small-group discussions and interactive problem solving allowed for immediate feedback about clinical reasoning skills as the case unfolded. Structuring case discussions to promote active engagement and critical thinking is essential for deep learning in clinical reasoning education.^18,24^ Integration of lectures and small-group activities also mitigates some of the challenges associated with small-group teaching (eg, facilitator shortage and lack of standardization among facilitators).

A close look at the pattern of results helps identify where learning was most meaningful. Confidence gains were largest in synthesizing diagnostic information and developing management plans, both of which require applied clinical reasoning. In comparison, improvement in constructing problem representations, although significant, was comparatively smaller, indicating that foundational summarization skills may benefit from additional practice. Among the 3 knowledge questions, improvement on the initial management of hematochezia was the smallest. This suggests that some learners may still struggle with early decision-making when diagnostic uncertainty is high. This is an opportunity for more explicit instruction or repeated practice in initial stabilization and diagnostic workup. The high levels of reported satisfaction and perceived improvement in clinical reasoning skills suggest that the interactive format of the case (intentionally designed to foster active engagement through multiple small-group problem-solving tasks) successfully engaged learners in articulating and practicing reasoning processes.

This study has several limitations. We assessed the impact of a single case embedded within a longitudinal course on clinical reasoning. Students had completed 8 similar sessions prior to the hematochezia case, making it difficult to know how prior practice impacted students’ experiences for this case. The generalizability of the impact of this single case outside the context of the course is limited. Additionally, we were only able to measure students’ self-assessment of their clinical reasoning skills, which may not reflect their actual skill level. Future studies that measure students’ clinical reasoning skills in workplace-based assessments are needed. The percentage of correct answers on the knowledge questions in the presurvey limit our ability to determine if the knowledge gained (as postsurvey correct answer percentages were higher for all 3 questions) was from the content we delivered or the interactivity of group members with each other, many of whom statistically knew the right answers.

The last limitation was the absence of more qualitative data. Specifically, we should have assessed which components of the case were effective in influencing clinical reasoning. Understanding how the students’ thinking shifted at each node of the case would also have been informative. Finally, there is a social component to clinical reasoning, and it would have been interesting to see how intragroup dynamics affected reasoning. The 2 most common responses to our qualitative item about shortening small-group discussion times and providing more small group faculty facilitators are helpful for others hoping to adapt our case for use in their own clinical reasoning courses.

Our exploratory pilot project supports the implementation of clinical reasoning instruction in preclinical medical education. Through structured, case-based learning, medical students’ confidence, attitudes, and knowledge improved. Extending medical education on clinical reasoning to preclinical settings is effective and feasible and should be considered as an educational strategy.

## Appendices


Hematochezia Case.pptxFacilitator Guide.docxClinical Reasoning Task Prompts.docxPresurvey.docxPostsurvey.docx
*All appendices are peer reviewed as integral parts of the Original Publication.*

## References

[1] Yazdani S, Hoseini Abardeh M. Five decades of research and theorization on clinical reasoning: a critical review. Adv Med Educ Pract. 2019;10:703–716. 10.2147/AMEP.S213492 31695548 PMC6717718

[2] Gruppen LD. Clinical reasoning: defining it, teaching it, assessing it, studying it. West J Emerg Med. 2017;18(1):4–7. 10.5811/westjem.2016.11.33191 28115999 PMC5226761

[3] Rencic J, Trowbridge RL Jr, Fagan M, Szauter K, Durning S. Clinical reasoning education at US medical schools: results from a national survey of internal medicine clerkship directors. J Gen Intern Med. 2017;32(11):1242–1246. 10.1007/s11606-017-4159-y 28840454 PMC5653563

[4] Parodis I, Andersson L, Durning SJ, et al. Clinical reasoning needs to be explicitly addressed in health professions curricula: recommendations from a European consortium. Int J Environ Res Public Health. 2021;18(21):11202. 10.3390/ijerph182111202 34769721 PMC8583438

[5] Richards JB, Hayes MM, Schwartzstein RM. Teaching clinical reasoning and critical thinking: from cognitive theory to practical application. Chest. 2020;158(4):1617–1628. 10.1016/j.chest.2020.05.525 32450242

[6] Gold JG, Knight CL, Christner JG, Mooney CE, Manthey DE, VJ Lang. Clinical reasoning education in the clerkship years: a cross-disciplinary national needs assessment. PLoS One. 2022;17(8):e0273250. 10.1371/journal.pone.0273250 35980994 PMC9387845

[7] Norman G, Pelaccia T, Wyer P, Sherbino J. Dual process models of clinical reasoning: the central role of knowledge in diagnostic expertise. J Eval Clin Pract. 2024;30(5):788–796. 10.1111/jep.13998 38825755

[8] Custers EJFM. Thirty years of illness scripts: theoretical origins and practical applications. Med Teach. 2015;37(5):457–462. 10.3109/0142159X.2014.956052 25180878

[9] Yardley S, Teunissen PW, Dornan T. Experiential learning: AMEE Guide No. 63. Med Teach. 2012;34(2):e102–e115. 10.3109/0142159X.2012.650741 22289008

[10] Kelekar A, Afonso N. Evaluation of the effect of a new clinical reasoning curriculum in a pre-clerkship clinical skills course. Perspect Med Educ. 2020;9(2):123–127. 10.1007/S40037-020-00566-4 32056123 PMC7138760

[11] Weinstein A, Pinto-Powell R. Introductory clinical reasoning curriculum. MedEdPORTAL. 2016;12:10370. 10.15766/mep_2374-8265.10370

[12] Chadha N, Fredrick D, Malbari A, Hojsak J. A virtual clinical reasoning case for medical students using an ophthalmology model: a case of red eye. MedEdPORTAL. 2021;17:11117. 10.15766/mep_2374-8265.11117 33768149 PMC7970637

[13] Thomas PA, Kern DE. Internet resources for curriculum development in medical education: an annotated bibliography. J Gen Intern Med. 2004;19:599–605. 10.1111/j.1525-1497.2004.99999.x 15109332 PMC1492314

[14] Thomas PA, Kern DE, Hughes MT, Tackett SA, Chen BY, eds. Curriculum Development for Medical Education: A Six-Step Approach. 4th ed. Johns Hopkins University Press; 2022.

[15] Artino AR Jr, La Rochelle JS, Dezee KJ, Gehlbach H. Developing questionnaires for educational research: AMEE Guide No. 87. Med Teach. 2014;36(6):463–474. 10.3109/0142159X.2014.889814 24661014 PMC4059192

[16] Kirkpatrick DL, Kirkpatrick JD. Evaluating Training Programs: The Four Levels. 3rd ed. Berrett-Koehler Publishers; 2006.

[17] Duca NS, Glod S. Bridging the gap between the classroom and the clerkship: a clinical reasoning curriculum for third-year medical students. MedEdPORTAL. 2019;15:10800. 10.15766/mep_2374-8265.10800 31139730 PMC6507921

[18] Ishizuka K, Shikino K, Takada N, et al. Enhancing clinical reasoning skills in medical students through team-based learning: a mixed-methods study. BMC Med Educ. 2025;25(1):221. 10.1186/s12909-025-06784-w 39934738 PMC11817391

[19] Staal J, Hooftman J, Gunput STG, et al. Effect on diagnostic accuracy of cognitive reasoning tools for the workplace setting: systematic review and meta-analysis. BMJ Qual Saf. 2022;31(12):899–910. 10.1136/bmjqs-2022-014865 PMC9685706 36396150

[20] Gottlieb M, Chan TM, Zaver F, Ellaway R. Confidence-competence alignment and the role of self-confidence in medical education: a conceptual review. Med Educ. 2022;56(1):37–47. 10.1111/medu.14592 34176144

[21] McNair R, Griffiths L, Reid K, Sloan H. Medical students developing confidence and patient centredness in diverse clinical settings: a longitudinal survey study. BMC Med Educ. 2016;16:176. 10.1186/s12909-016-0689-y 27421655 PMC4946086

[22] Delavari S, Barzkar F, Rikers MJPR, et al. Teaching and learning clinical reasoning skill in undergraduate medical students: a scoping review. PLoS One. 2024;19(10):e0309606. 10.1371/journal.pone.0309606 39413083 PMC11482728

[23] Gruppen LD. Outcome-based medical education: implications, opportunities, and challenges. Korean J Med Educ. 2012;24(4):281–285. 10.3946/kjme.2012.24.4.281 25813324 PMC8813364

[24] Levin M, Cennimo D, Chen S, Lamba S. Teaching clinical reasoning to medical students: a case-based illness script worksheet approach. MedEdPORTAL. 2016;12:10445. 10.15766/mep_2374-8265.10445 31008223 PMC6464440

