# Supplementary material for: The Development and Evaluation of a Clinical Reasoning Case for Second-Year Medical Students
Source: MedEdPORTAL. 2026 Apr 28;22:11596. doi: 10.15766/mep_2374-8265.11596 (PMC13123434; doi:10.15766/mep_2374-8265.11596)
Supplement: Supplementary file 1 — Hematochezia Case.pptxFacilitator Guide.docxClinical Reasoning Task Prompts.docxPresurvey.docxPostsurvey.docx [file mep_2374-8265.11596-s001.zip › B. Facilitator Guide.docx]

**Faculty Guide: Interactive Clinical Reasoning Session – Crohn’s Disease**

**Overview**

This faculty guide accompanies an interactive, case-based PowerPoint presentation designed for pre-clinical and early clinical medical students. The session uses progressive case disclosure (aliquots) to simulate real-time clinical reasoning in the evaluation of gastrointestinal bleeding and inflammatory bowel disease (IBD). Students collaborate in small groups to complete reasoning tasks and then reconvene for facilitated large-group debriefs led by the instructor.

**Learning Objectives**

By the end of the session, learners will be able to:

1. Construct a problem representation synthesizing key clinical data.
2. Develop a prioritized differential diagnosis for hematochezia.
3. Differentiate between upper and lower gastrointestinal bleeding using clinical clues.
4. Identify clinical features distinguishing Crohn’s disease from ulcerative colitis.
5. Interpret laboratory, imaging, and endoscopic findings in suspected IBD.
6. Propose an initial management and monitoring plan for a patient with Crohn’s disease.

**Instructional Logistics**

**Audience & Setting**: This session is designed for a large group of medical students and is best delivered in a lecture hall or auditorium equipped with a projector. Students should be seated in clusters (~8–10 per group) to facilitate small-group interaction.

**Format**: The session alternates between:

- **Peach slides**: Case aliquots (history, physical exam, labs, imaging, etc.)
- **Dark blue slides**: Small-group clinical reasoning tasks
- **White slides**: Faculty-led teaching and debriefing

**Facilitation Tips**:

- While small groups do not require embedded facilitators, adding them can enrich group dynamics.
- Clinical reasoning tasks can be distributed via printed handouts or collaborative digital platforms.
- Students may use online resources but should avoid AI tools such as ChatGPT to preserve the integrity of group reasoning and discourse.
- The total time required for this activity is 2 hours.

**Slide-by-Slide Faculty Instructions**

**Slide 1 – Introduction**

- Explain the progressive case disclosure format and session objectives.
- Emphasize the alternation between group work and faculty debrief.

**Slide 2 – Chief Concern**

- Read aloud the chief complaint to the class.
- Set the stage for hypothesis-driven history-taking.

**Slide 3 – Group Task #1 (10 min)**

- Prompt students to formulate history questions using a hypothesis-driven approach.
- Encourage each group to record and prioritize key questions.

**Slides 4–8 – History & Physical Exam**

- Present the information step-by-step.
- Distribute printed copies if desired (excluding future slides).
- Ask students to note pertinent positives/negatives.

**Slide 9 – Group Task #2 (15 min)**

- Ask each group to:
  - Develop a concise problem representation.
  - Differentiate upper vs. lower GI bleeding.
  - List top 3 differential diagnoses and propose an initial work-up, including rationale for diagnostic tests.

**Slides 10–16 – Debriefing Task #2**

- Walk through an expert-level approach:
  - Slide 10: Problem representation
  - Slide 11–13: Rectal bleeding and melena vs. hematochezia
  - Slide 14–15: Structured approach to hematochezia
  - Slide 16: Recommended labs and rationale

**Slides 17–21 – Additional Case Data**

- Present lab and imaging results.
- Encourage reflection on how the new data informs diagnostic reasoning.

**Slide 22 – Group Task #3 (15 min)**

- Students refine their problem representation and differential using new data.
- Encourage students to justify updates and consider test performance characteristics.

**Slides 23–26 – Debriefing Task #3**

- Demonstrate interpretation of new data.
- Highlight changes to the differential and next diagnostic steps.

**Slide 27 – Additional Case Data**

- Reveal serologic/stool testing and colonoscopy results.

**Slide 28 – Group Task #4**

- Groups update their clinical impressions, diagnoses, and initial management plan.

**Slides 29–30 – Debriefing Task #4**

- Present updated expert-level problem representation and diagnosis.
- Discuss rationale for diagnosis and initial treatment plan.

**Slides 31–32 – Group Task #5**

- Groups compare and contrast Crohn’s disease vs. ulcerative colitis:
  - Epidemiology
  - Clinical features
  - Diagnostics
  - Management
- Use the framework on slide 32 to build a management plan.

**Slides 33–36 – Debriefing Task #5**

- Review key comparisons between Crohn’s and UC.
- Discuss evidence-based treatment strategies.
- Conclude with an illness script summarizing Crohn’s disease (slide 36).
