# Supplementary material for: The Development and Evaluation of a Clinical Reasoning Case for Second-Year Medical Students
Source: MedEdPORTAL. 2026 Apr 28;22:11596. doi: 10.15766/mep_2374-8265.11596 (PMC13123434; doi:10.15766/mep_2374-8265.11596)
Supplement: Supplementary file 1 — Hematochezia Case.pptxFacilitator Guide.docxClinical Reasoning Task Prompts.docxPresurvey.docxPostsurvey.docx [file mep_2374-8265.11596-s001.zip › C. Clinical Reasoning Task Prompts.docx]

Task #1 (10 minutes):

Discuss with your group and write down history questions (HPI, PMH, Meds, ALL, SH, FH) you would like to ask this patient to help you figure out what's going on and what next steps you want to take.

Task #2 (15 minutes):

Write a problem representation for the patient's presenting symptoms:

Write down what presenting symptoms the patient has that would point you towards the source of bleeding being from the upper vs lower GI tract:

Write a prioritized differential diagnosis for the patient's symptoms:

Write a list of studies you would like to order:

Task #3 (15 minutes)

Write out your interpretation of the labs (pertinent positives and negatives)

What immediate management interventions are needed?

- - How would this be different if the hemoglobin was lower (6.3) and/or the patient was light-headed?

Update your problem representation

Write a list of additional studies you would like to order

Task #4 (5 minutes):

Update your problem representation and prioritized differential diagnosis with the new lab and colonoscopy data

Task #5 (10 minutes)

Compare and contrast Crohn's Disease and Ulcerative Colitis using illness scripts

Create a preliminary treatment plan for this patient
