# Supplementary material for: The Development and Evaluation of a Clinical Reasoning Case for Second-Year Medical Students
Source: MedEdPORTAL. 2026 Apr 28;22:11596. doi: 10.15766/mep_2374-8265.11596 (PMC13123434; doi:10.15766/mep_2374-8265.11596)
Supplement: Supplementary file 1 — Hematochezia Case.pptxFacilitator Guide.docxClinical Reasoning Task Prompts.docxPresurvey.docxPostsurvey.docx [file mep_2374-8265.11596-s001.zip › D. Presurvey.docx]

ISP Pre-survey

Start of Block: Pre-Survey

Disclaimer You are being invited to participate in a research study titled “Effectiveness of Formally Implementing Clinical Reasoning Training during Pre-Clinical Medical Education: Insights Through a Small-Group Case on Hematochezia,” focused on medical students' understanding of clinical reasoning application to a patient case on hematochezia. This study is being done by Dr. William McGuire and medical student Millie Kirchberg from UC San Diego. You were selected to participate in this study because you are a second-year medical student enrolled in the Clinical Decision-Making course at UCSD SOM. The purpose of this research study is to evaluate the effectiveness of formally teaching and applying clinical reasoning skills prior to beginning third year clerkships. This project seeks to explore whether this session improves students' confidence, attitudes, and knowledge regarding their clinical reasoning skills. Your participation in this research should last approximately 20-30 minutes. If you agree to take part in this study, you will be asked to complete a pre-session and post-session online survey through Qualtrics. These surveys will assess your knowledge of hematochezia, your confidence with utilizing clinical reasoning skills, and your attitudes regarding implementing clinical reasoning training into the curriculum, both before and after completing the case. The surveys will take approximately 10 minutes each to complete. Your participation in this study is completely voluntary and you can withdraw at any time. Choosing not to participate or withdrawing will result in no penalty or loss of benefits to which you are entitled. You are free to skip any question that you choose. If you have questions about this project or if you have a research-related problem, you may contact the researcher(s), principal investigator at wmcguire@health.ucsd.edu or co-investigator at mkirchberg@health.ucsd.edu. If you have any questions concerning your rights as a research subject, you may contact the UC San Diego Office of IRB Administration at irb@ucsd.edu or 858-246-4777. By participating in this research you are indicating that you are at least 18 years old, have read this consent form, and agree to participate in this research study. Please keep this consent form for your records.

Q1 Please type your first and last name

________________________________________________________________

Confidence Questions Q2:

Q2 How confident are you with the following?

|  | Extremely Unconfident (1) | Moderately Unconfident (2) | Slightly Unconfident (3) | Neither confident nor unconfident (4) | Slightly Confident (5) | Moderately Confident (6) | Extremely Confident (7) |
| --- | --- | --- | --- | --- | --- | --- | --- |
| Writing a problem representation (1) |  |  |  |  |  |  |  |
| Creating a differential diagnosis for hematochezia (2) |  |  |  |  |  |  |  |
| Creating an illness script for hematochezia (3) |  |  |  |  |  |  |  |
| Initial lab and imaging work-up for hematochezia (4) |  |  |  |  |  |  |  |
| Ability to interpret the results of diagnostic tests related to hematochezia (5) |  |  |  |  |  |  |  |
| Creating a treatment plan for the underlying cause of hematochezia (6) |  |  |  |  |  |  |  |

Attitudes Questions Q3-4:

Q3 What are your attitudes regarding the following questions?

|  | Not valuable (1) | Slightly valuable (2) | Moderately valuable (3) | Very valuable (4) | Extremely valuable (5) |
| --- | --- | --- | --- | --- | --- |
| How valuable do you find case-based learning exercises in enhancing your clinical reasoning for hematochezia? (1) |  |  |  |  |  |
| How valuable do you find clinical reasoning cases are for preparing you for clerkships? (2) |  |  |  |  |  |
| How valuable do you find a small-group format is compared to individual work for clinical reasoning cases? (3) |  |  |  |  |  |

Q4 Which aspects of clinical reasoning do you find most challenging when dealing with hematochezia? (Select all that apply)

- Differentiating between possible causes (1)
- Identifying appropriate diagnostic tests (2)
- Integrating patient history and physical examination findings (3)
- Developing a differential diagnosis (4)
- Other (please specify): (5) __________________________________________________

Knowledge Questions Q5-7:

Q5 Which of the following conditions is NOT a common cause of hematochezia?

- Colorectal cancer (1)
- Peptic ulcer disease (2)
- Hemorrhoids (3)
- Inflammatory bowel disease (4)
- Diverticulosis (5)

Q6 What initial step should be taken when a patient presents with hematochezia?

- Comprehensive history and physical examination (1)
- Immediate endoscopy (2)
- Prescribing an antidiarrheal medication (3)
- Ordering a CT scan (4)
- Starting a blood transfusion (5)

Q7 What is a key difference in the clinical approach between a patient with hematochezia and a patient with melena?

- Hematochezia is typically less severe than melena. (1)
- Hematochezia usually indicates lower gastrointestinal bleeding, while melena indicates upper gastrointestinal bleeding. (2)
- Both conditions require the same diagnostic approach. (3)
- Melena is associated with a higher risk of malignancy than hematochezia. (4)

End of Block: Pre-Survey
