# Supplementary material for: The Development and Evaluation of a Clinical Reasoning Case for Second-Year Medical Students
Source: MedEdPORTAL. 2026 Apr 28;22:11596. doi: 10.15766/mep_2374-8265.11596 (PMC13123434; doi:10.15766/mep_2374-8265.11596)
Supplement: Supplementary file 1 — Hematochezia Case.pptxFacilitator Guide.docxClinical Reasoning Task Prompts.docxPresurvey.docxPostsurvey.docx [file mep_2374-8265.11596-s001.zip › A. Hematochezia Case.pptx]

## Slide 1
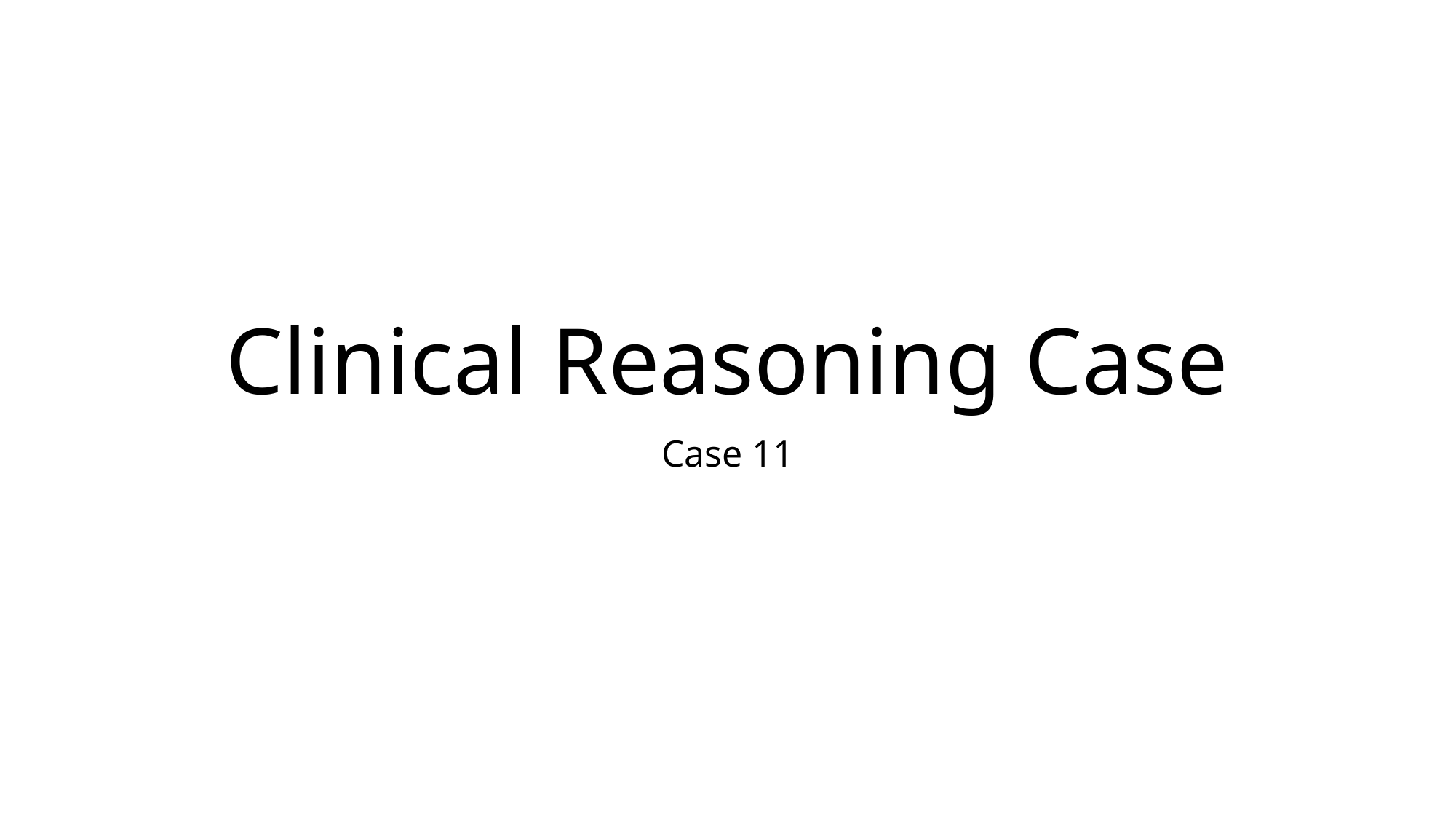

# Clinical Reasoning Case
Case 11

## Slide 2
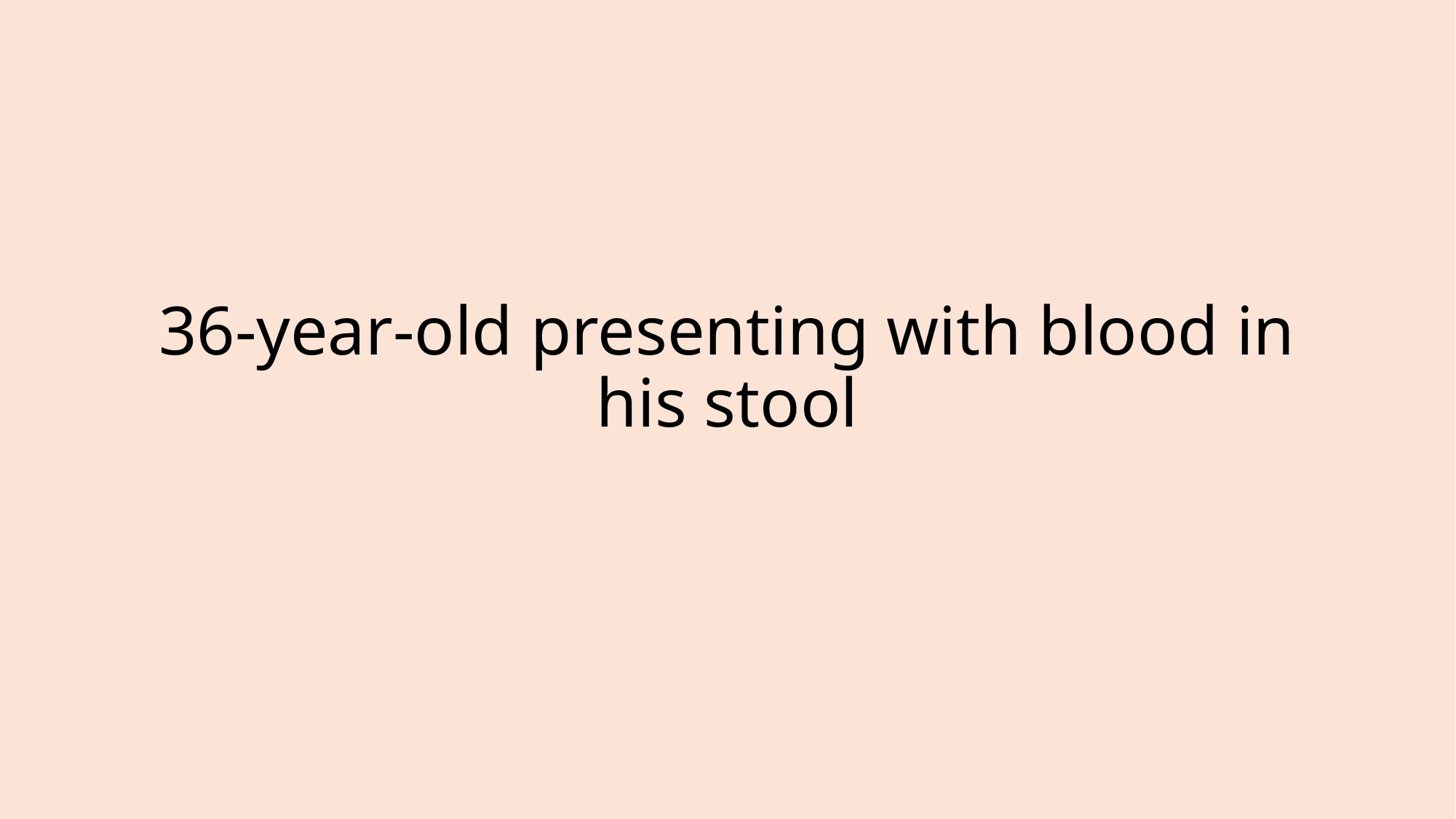

# 36-year-old presenting with blood in his stool

## Slide 3
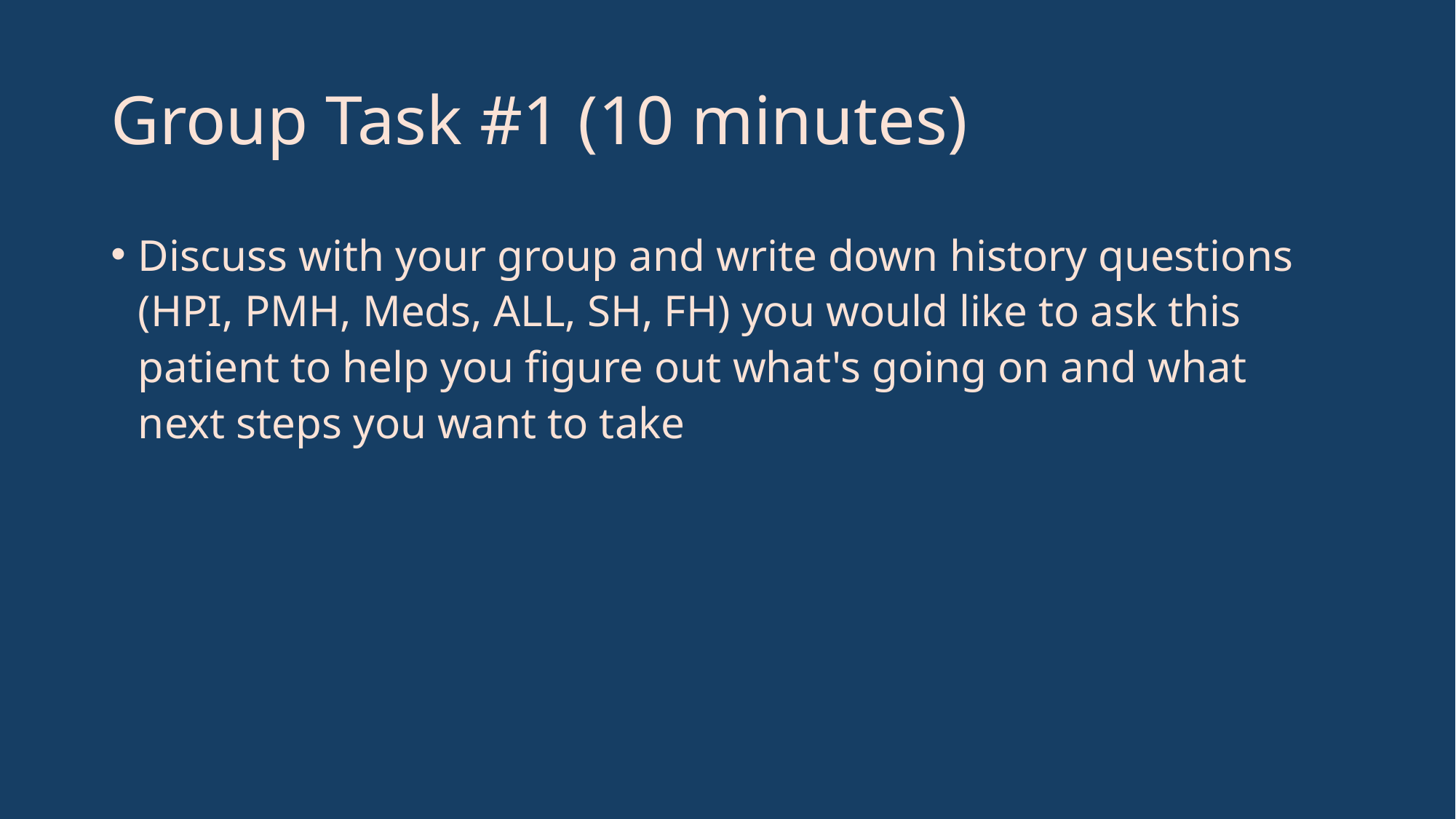

# Group Task #1 (10 minutes)
Discuss with your group and write down history questions (HPI, PMH, Meds, ALL, SH, FH) you would like to ask this patient to help you figure out what's going on and what next steps you want to take

## Slide 4
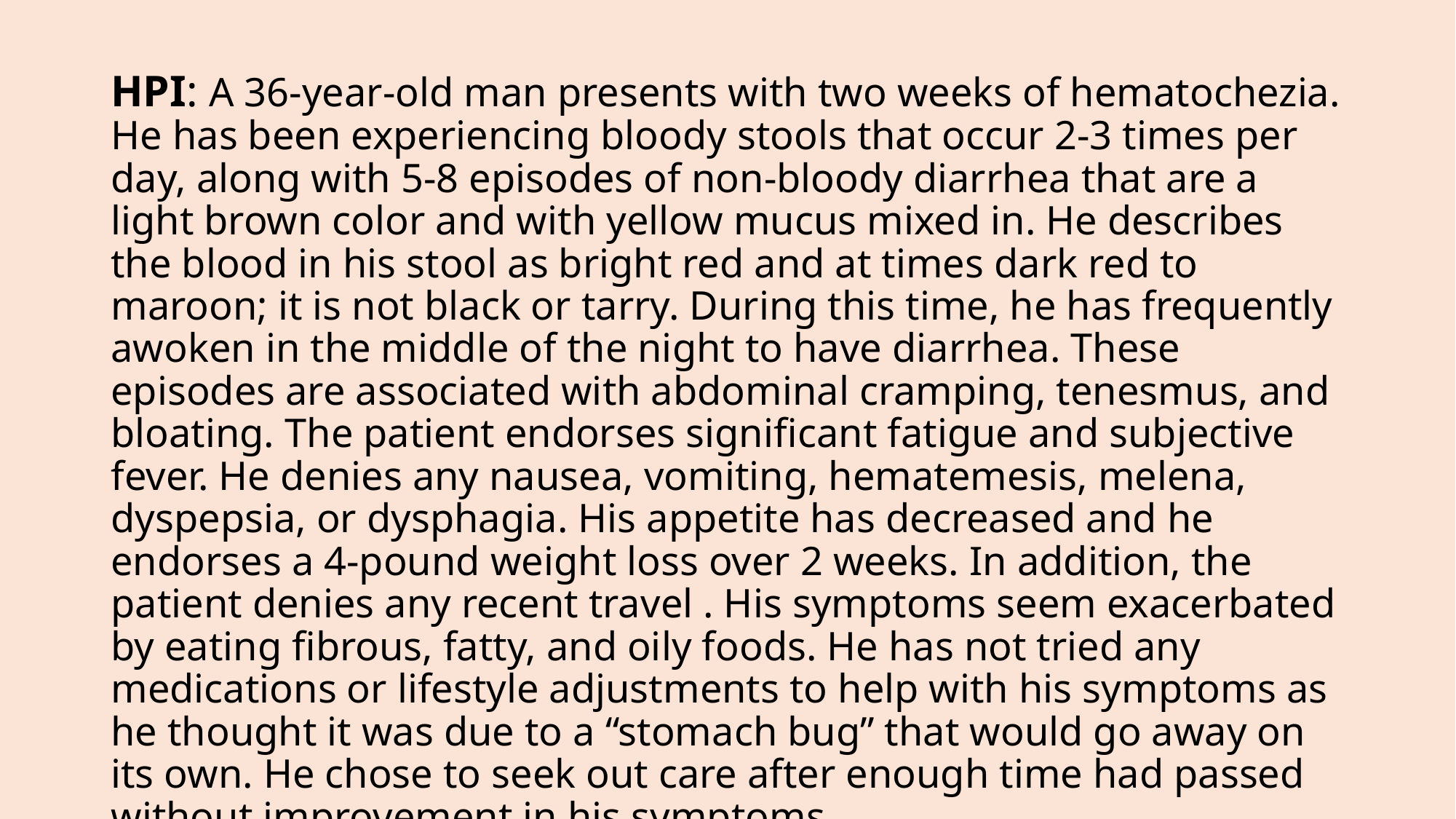

HPI: A 36-year-old man presents with two weeks of hematochezia. He has been experiencing bloody stools that occur 2-3 times per day, along with 5-8 episodes of non-bloody diarrhea that are a light brown color and with yellow mucus mixed in. He describes the blood in his stool as bright red and at times dark red to maroon; it is not black or tarry. During this time, he has frequently awoken in the middle of the night to have diarrhea. These episodes are associated with abdominal cramping, tenesmus, and bloating. The patient endorses significant fatigue and subjective fever. He denies any nausea, vomiting, hematemesis, melena, dyspepsia, or dysphagia. His appetite has decreased and he endorses a 4-pound weight loss over 2 weeks. In addition, the patient denies any recent travel . His symptoms seem exacerbated by eating fibrous, fatty, and oily foods. He has not tried any medications or lifestyle adjustments to help with his symptoms as he thought it was due to a “stomach bug” that would go away on its own. He chose to seek out care after enough time had passed without improvement in his symptoms.

## Slide 5
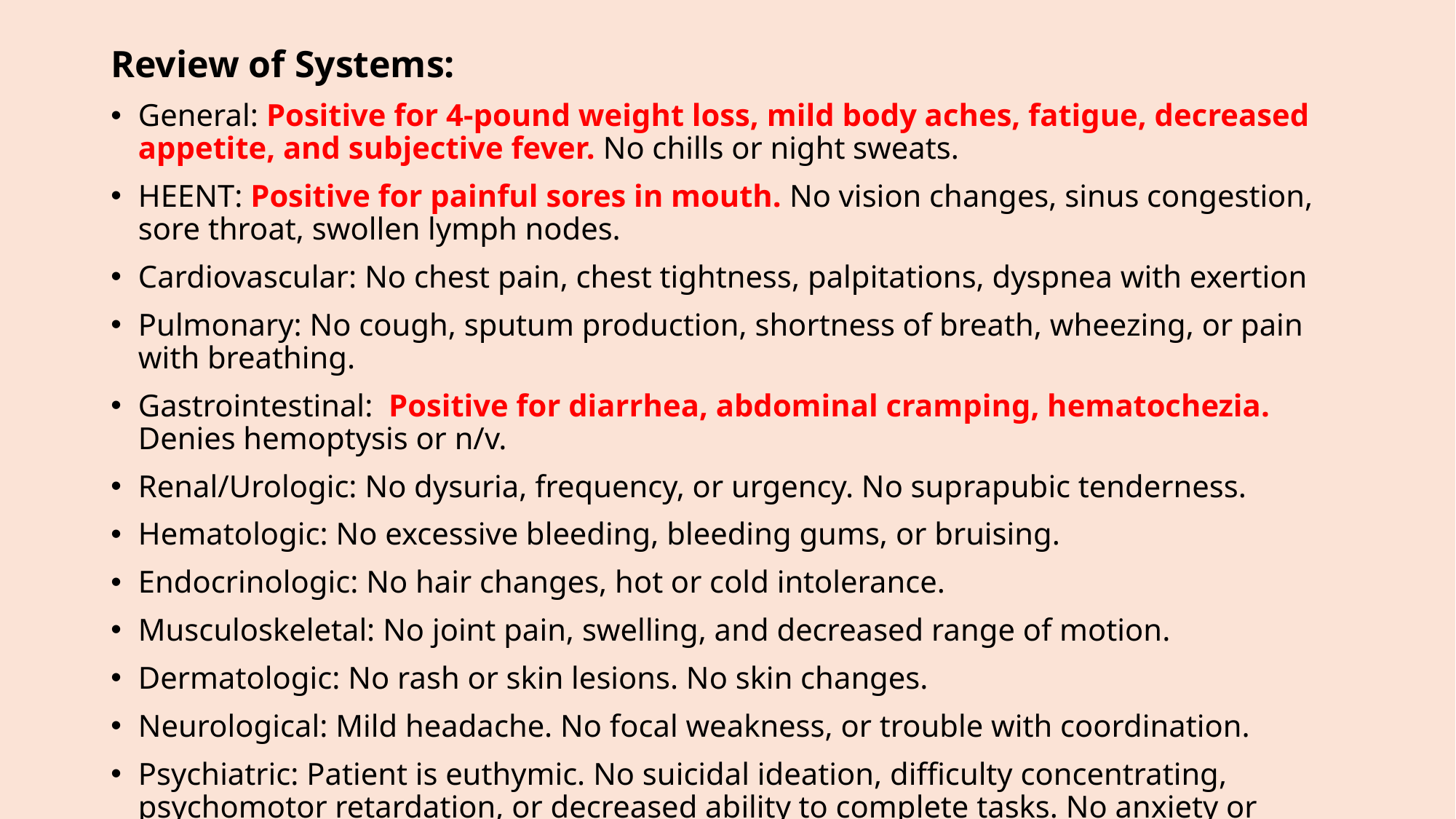

Review of Systems:
General: Positive for 4-pound weight loss, mild body aches, fatigue, decreased appetite, and subjective fever. No chills or night sweats.
HEENT: Positive for painful sores in mouth. No vision changes, sinus congestion, sore throat, swollen lymph nodes.
Cardiovascular: No chest pain, chest tightness, palpitations, dyspnea with exertion
Pulmonary: No cough, sputum production, shortness of breath, wheezing, or pain with breathing.
Gastrointestinal:  Positive for diarrhea, abdominal cramping, hematochezia. Denies hemoptysis or n/v.
Renal/Urologic: No dysuria, frequency, or urgency. No suprapubic tenderness.
Hematologic: No excessive bleeding, bleeding gums, or bruising.
Endocrinologic: No hair changes, hot or cold intolerance.
Musculoskeletal: No joint pain, swelling, and decreased range of motion.
Dermatologic: No rash or skin lesions. No skin changes.
Neurological: Mild headache. No focal weakness, or trouble with coordination.
Psychiatric: Patient is euthymic. No suicidal ideation, difficulty concentrating, psychomotor retardation, or decreased ability to complete tasks. No anxiety or sleep changes.

## Slide 6
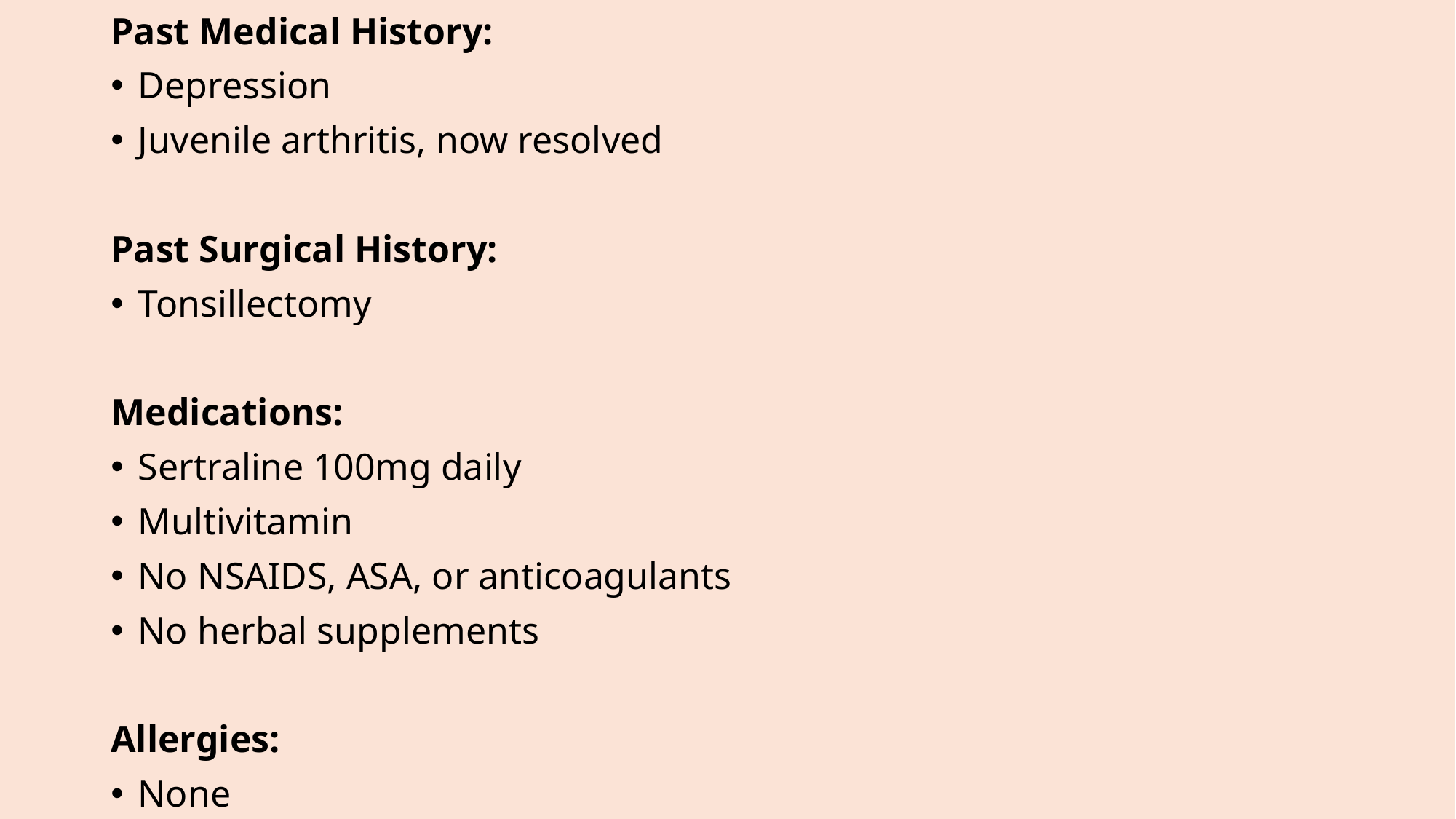

Past Medical History:
Depression
Juvenile arthritis, now resolved
Past Surgical History:
Tonsillectomy
Medications:
Sertraline 100mg daily
Multivitamin
No NSAIDS, ASA, or anticoagulants
No herbal supplements
Allergies:
None

## Slide 7
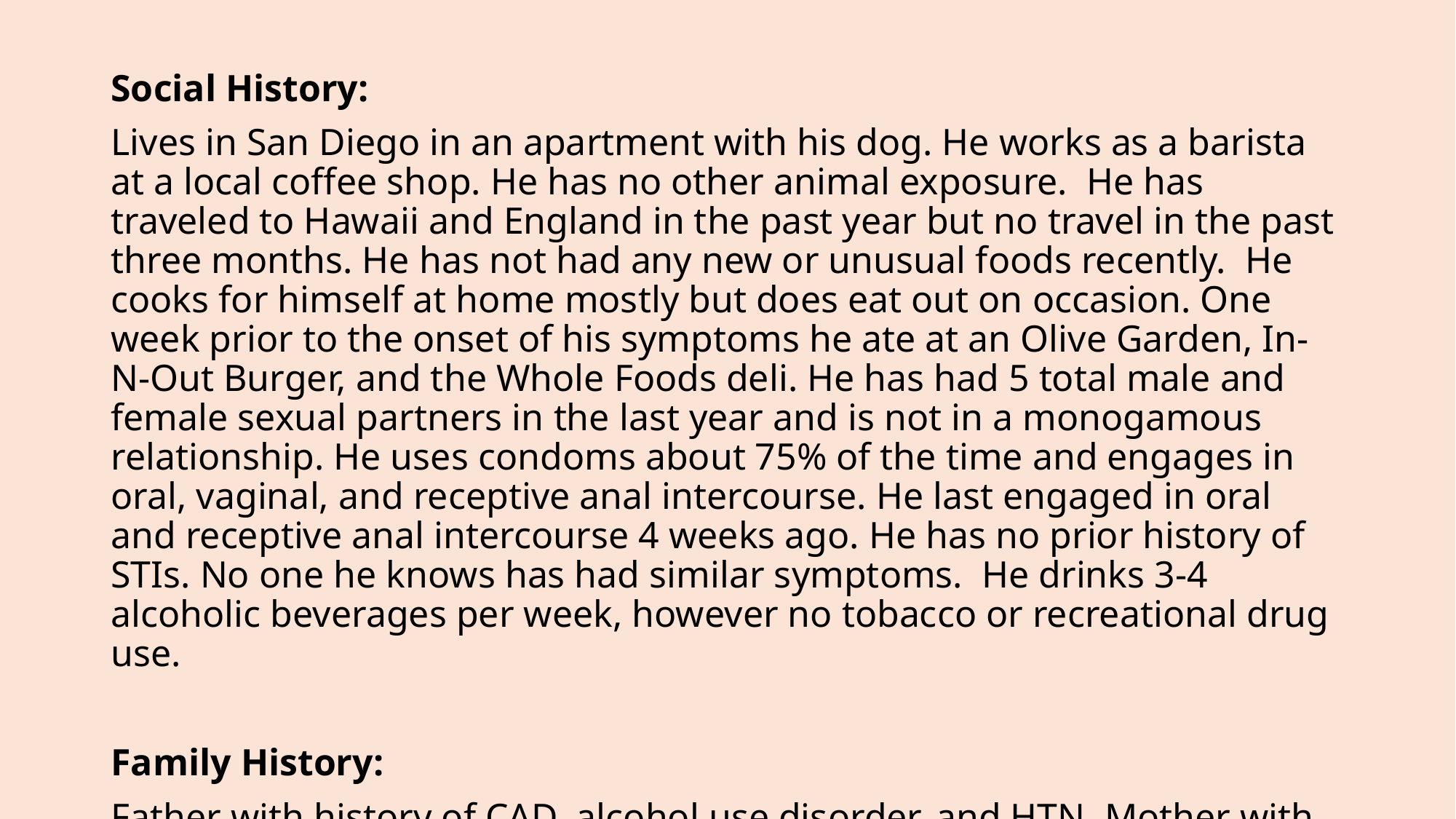

Social History:
Lives in San Diego in an apartment with his dog. He works as a barista at a local coffee shop. He has no other animal exposure.  He has traveled to Hawaii and England in the past year but no travel in the past three months. He has not had any new or unusual foods recently.  He cooks for himself at home mostly but does eat out on occasion. One week prior to the onset of his symptoms he ate at an Olive Garden, In-N-Out Burger, and the Whole Foods deli. He has had 5 total male and female sexual partners in the last year and is not in a monogamous relationship. He uses condoms about 75% of the time and engages in oral, vaginal, and receptive anal intercourse. He last engaged in oral and receptive anal intercourse 4 weeks ago. He has no prior history of STIs. No one he knows has had similar symptoms.  He drinks 3-4 alcoholic beverages per week, however no tobacco or recreational drug use.
Family History:
Father with history of CAD, alcohol use disorder, and HTN. Mother with history of T2DM and HLD. Younger brother with eczema.

## Slide 8
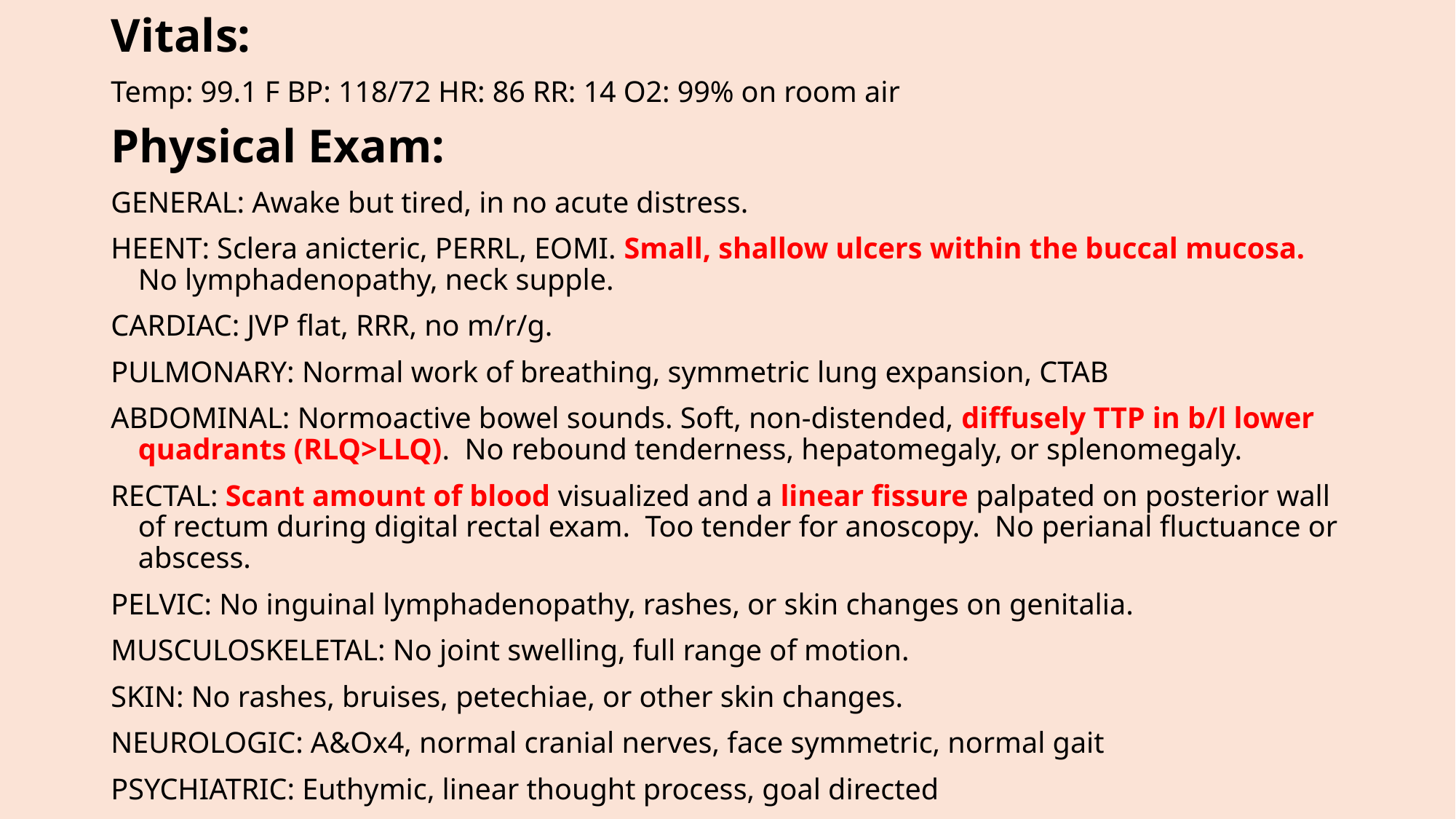

Vitals:
Temp: 99.1 F BP: 118/72 HR: 86 RR: 14 O2: 99% on room air
Physical Exam:
GENERAL: Awake but tired, in no acute distress.
HEENT: Sclera anicteric, PERRL, EOMI. Small, shallow ulcers within the buccal mucosa. No lymphadenopathy, neck supple.
CARDIAC: JVP flat, RRR, no m/r/g.
PULMONARY: Normal work of breathing, symmetric lung expansion, CTAB
ABDOMINAL: Normoactive bowel sounds. Soft, non-distended, diffusely TTP in b/l lower quadrants (RLQ>LLQ).  No rebound tenderness, hepatomegaly, or splenomegaly.
RECTAL: Scant amount of blood visualized and a linear fissure palpated on posterior wall of rectum during digital rectal exam.  Too tender for anoscopy.  No perianal fluctuance or abscess.
PELVIC: No inguinal lymphadenopathy, rashes, or skin changes on genitalia.
MUSCULOSKELETAL: No joint swelling, full range of motion.
SKIN: No rashes, bruises, petechiae, or other skin changes.
NEUROLOGIC: A&Ox4, normal cranial nerves, face symmetric, normal gait
PSYCHIATRIC: Euthymic, linear thought process, goal directed

## Slide 9
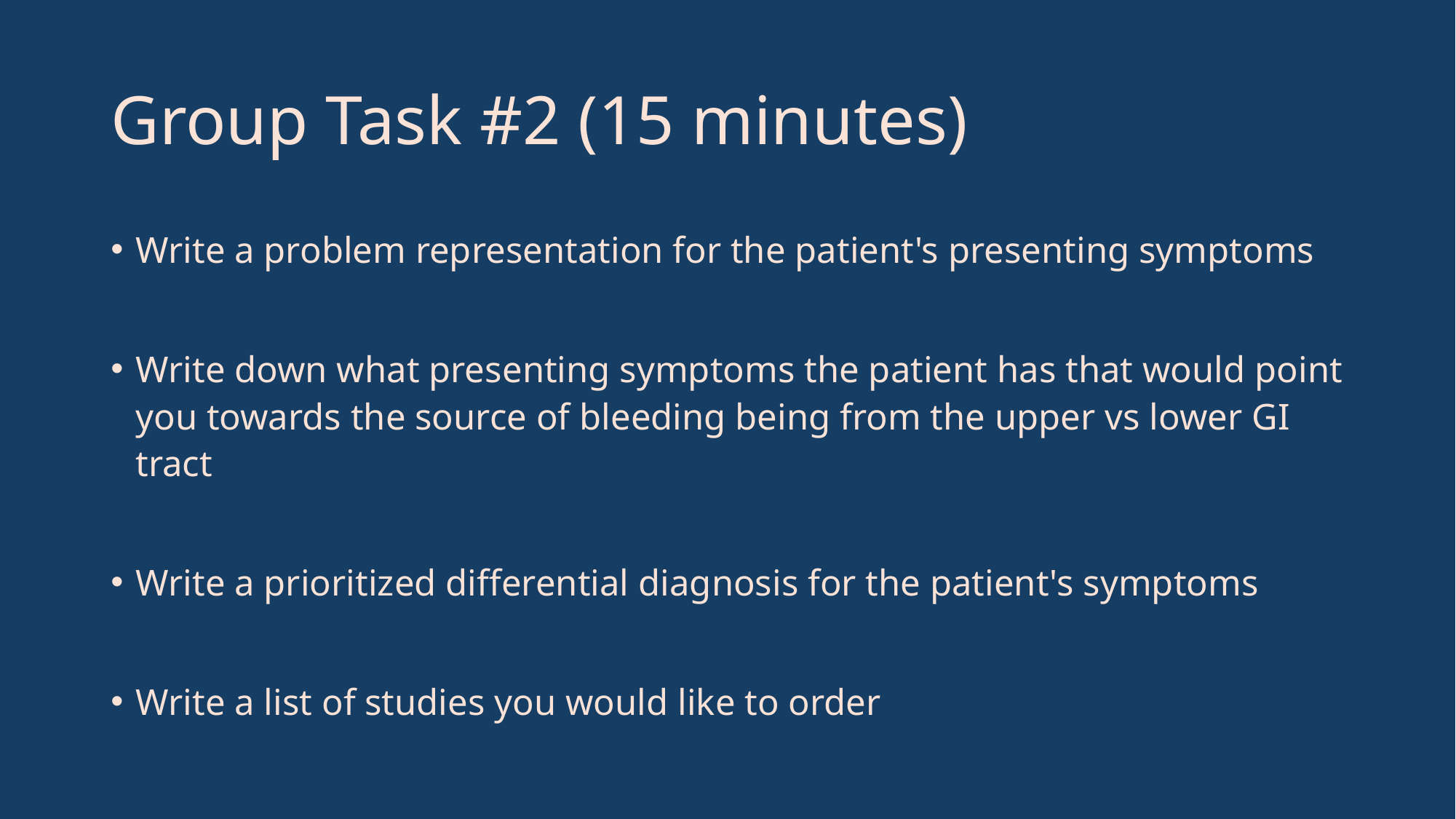

# Group Task #2 (15 minutes)
Write a problem representation for the patient's presenting symptoms
Write down what presenting symptoms the patient has that would point you towards the source of bleeding being from the upper vs lower GI tract
Write a prioritized differential diagnosis for the patient's symptoms
Write a list of studies you would like to order

## Slide 10
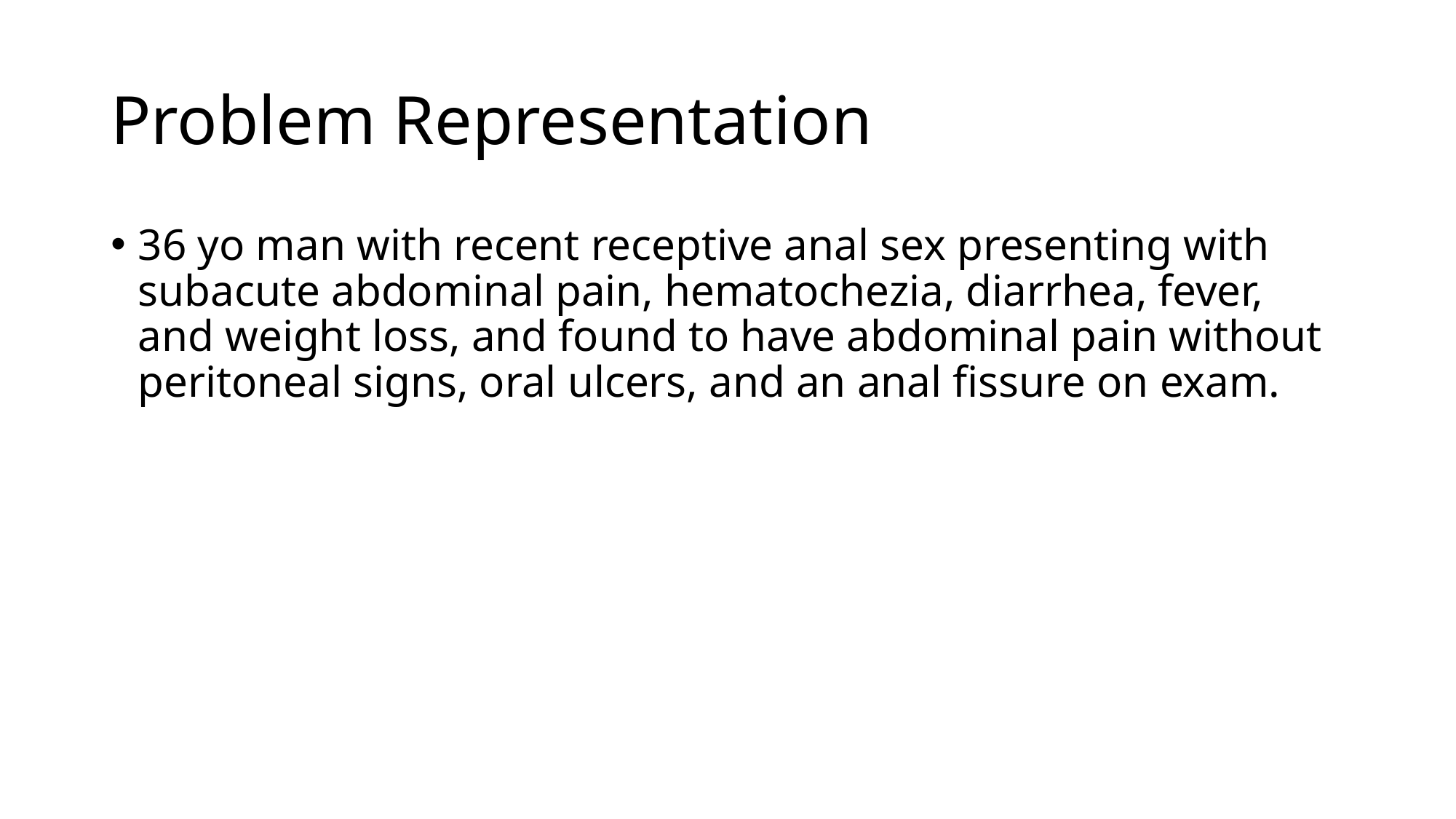

# Problem Representation
36 yo man with recent receptive anal sex presenting with subacute abdominal pain, hematochezia, diarrhea, fever, and weight loss, and found to have abdominal pain without peritoneal signs, oral ulcers, and an anal fissure on exam.

## Slide 11
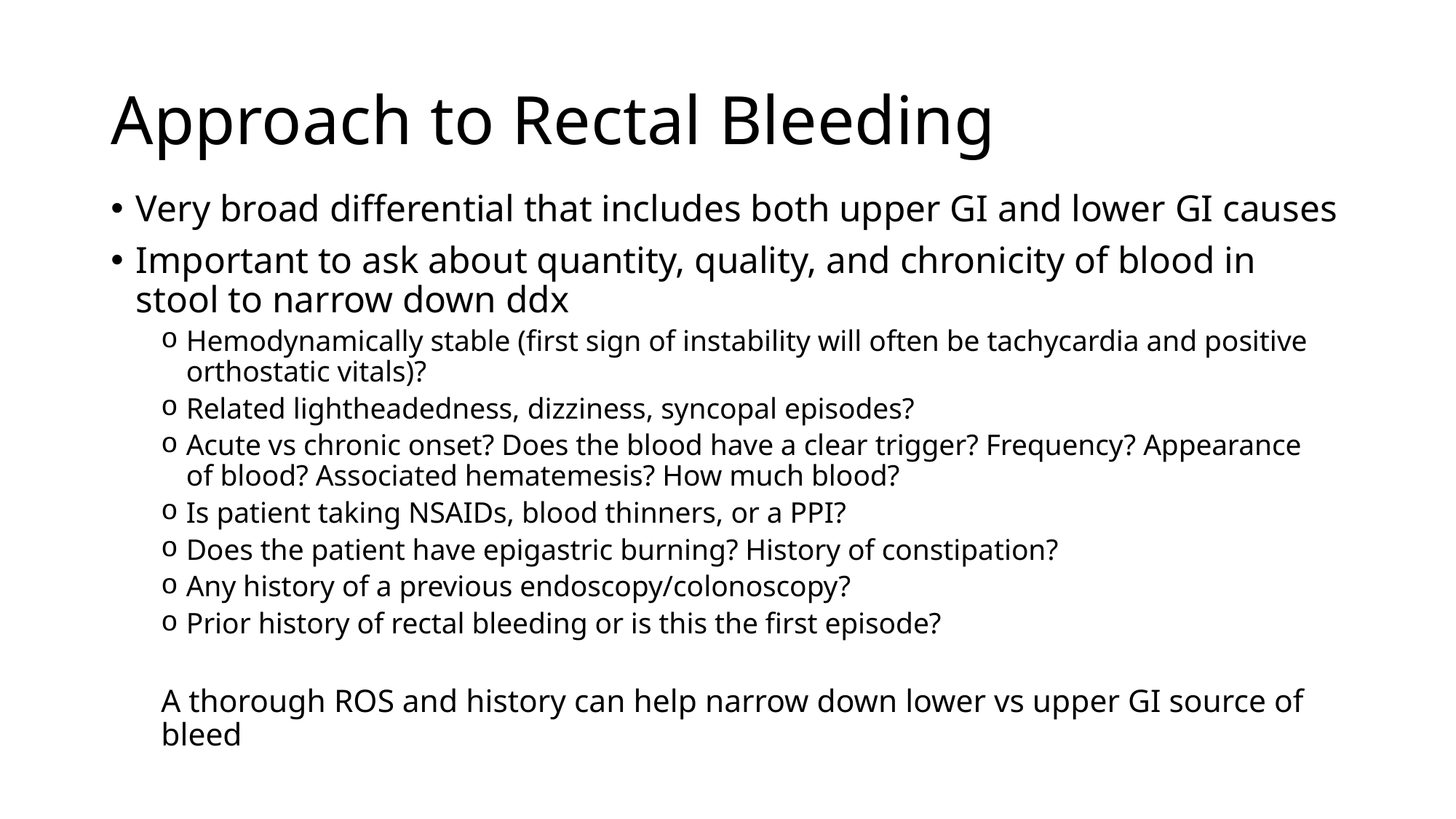

# Approach to Rectal Bleeding
Very broad differential that includes both upper GI and lower GI causes
Important to ask about quantity, quality, and chronicity of blood in stool to narrow down ddx
Hemodynamically stable (first sign of instability will often be tachycardia and positive orthostatic vitals)?
Related lightheadedness, dizziness, syncopal episodes?
Acute vs chronic onset? Does the blood have a clear trigger? Frequency? Appearance of blood? Associated hematemesis? How much blood?
Is patient taking NSAIDs, blood thinners, or a PPI?
Does the patient have epigastric burning? History of constipation?
Any history of a previous endoscopy/colonoscopy?
Prior history of rectal bleeding or is this the first episode?
A thorough ROS and history can help narrow down lower vs upper GI source of bleed

## Slide 12
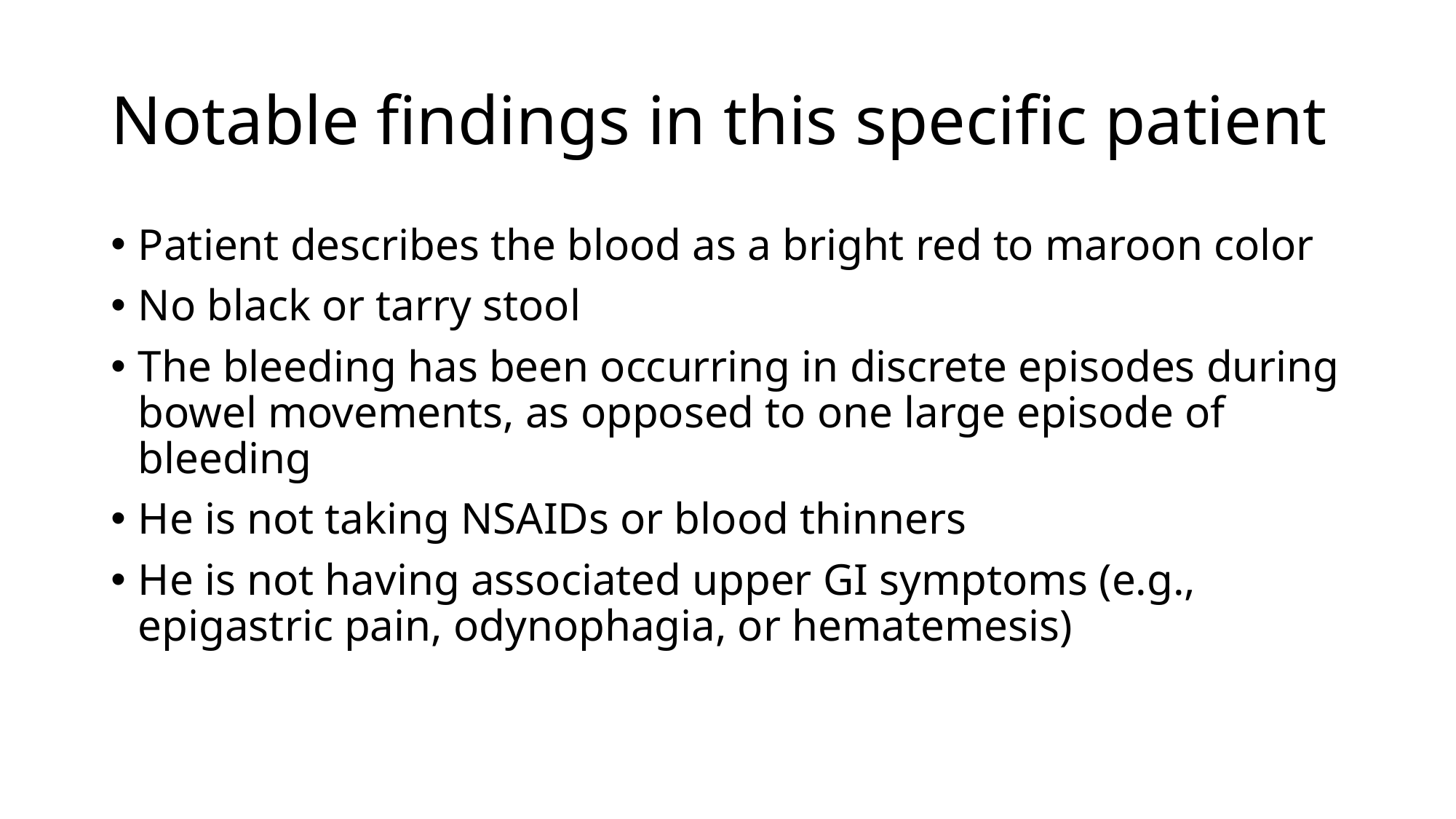

# Notable findings in this specific patient
Patient describes the blood as a bright red to maroon color
No black or tarry stool
The bleeding has been occurring in discrete episodes during bowel movements, as opposed to one large episode of bleeding
He is not taking NSAIDs or blood thinners
He is not having associated upper GI symptoms (e.g., epigastric pain, odynophagia, or hematemesis)

## Slide 13
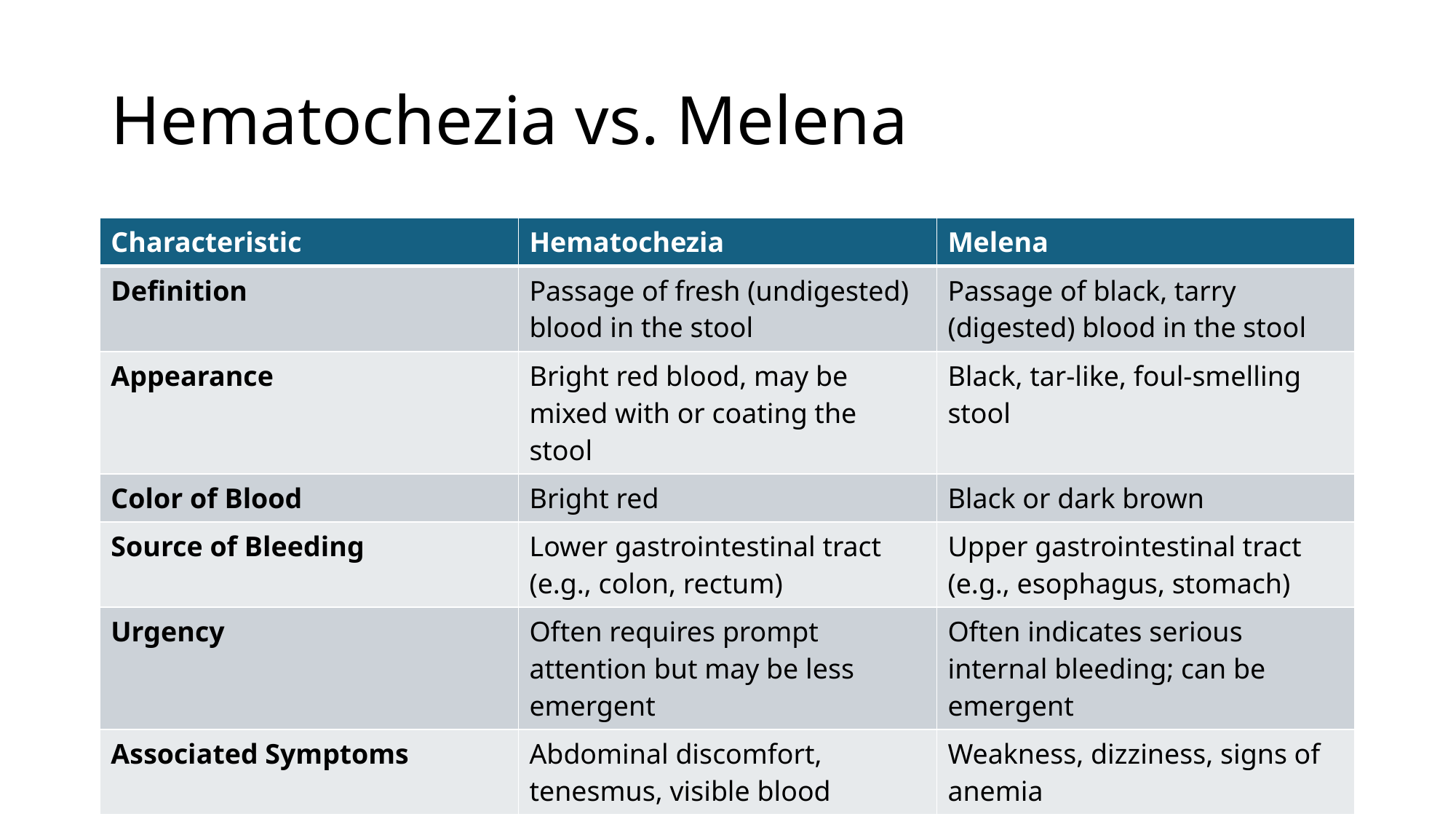

# Hematochezia vs. Melena
| Characteristic | Hematochezia | Melena |
| --- | --- | --- |
| Definition | Passage of fresh (undigested) blood in the stool | Passage of black, tarry (digested) blood in the stool |
| Appearance | Bright red blood, may be mixed with or coating the stool | Black, tar-like, foul-smelling stool |
| Color of Blood | Bright red | Black or dark brown |
| Source of Bleeding | Lower gastrointestinal tract (e.g., colon, rectum) | Upper gastrointestinal tract (e.g., esophagus, stomach) |
| Urgency | Often requires prompt attention but may be less emergent | Often indicates serious internal bleeding; can be emergent |
| Associated Symptoms | Abdominal discomfort, tenesmus, visible blood | Weakness, dizziness, signs of anemia |

## Slide 14
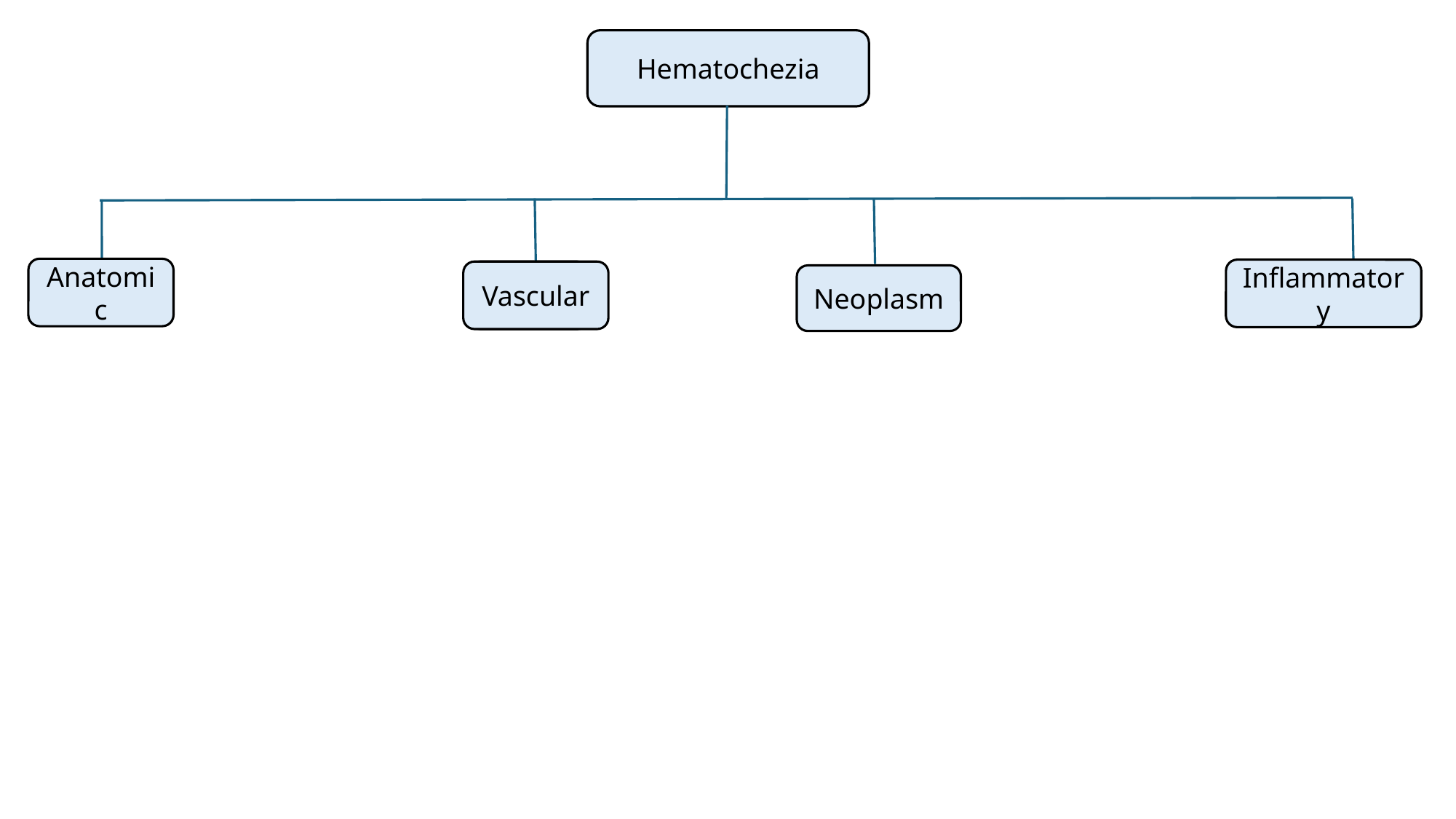

Hematochezia
Anatomic
Inflammatory
Vascular
Neoplasm

## Slide 15
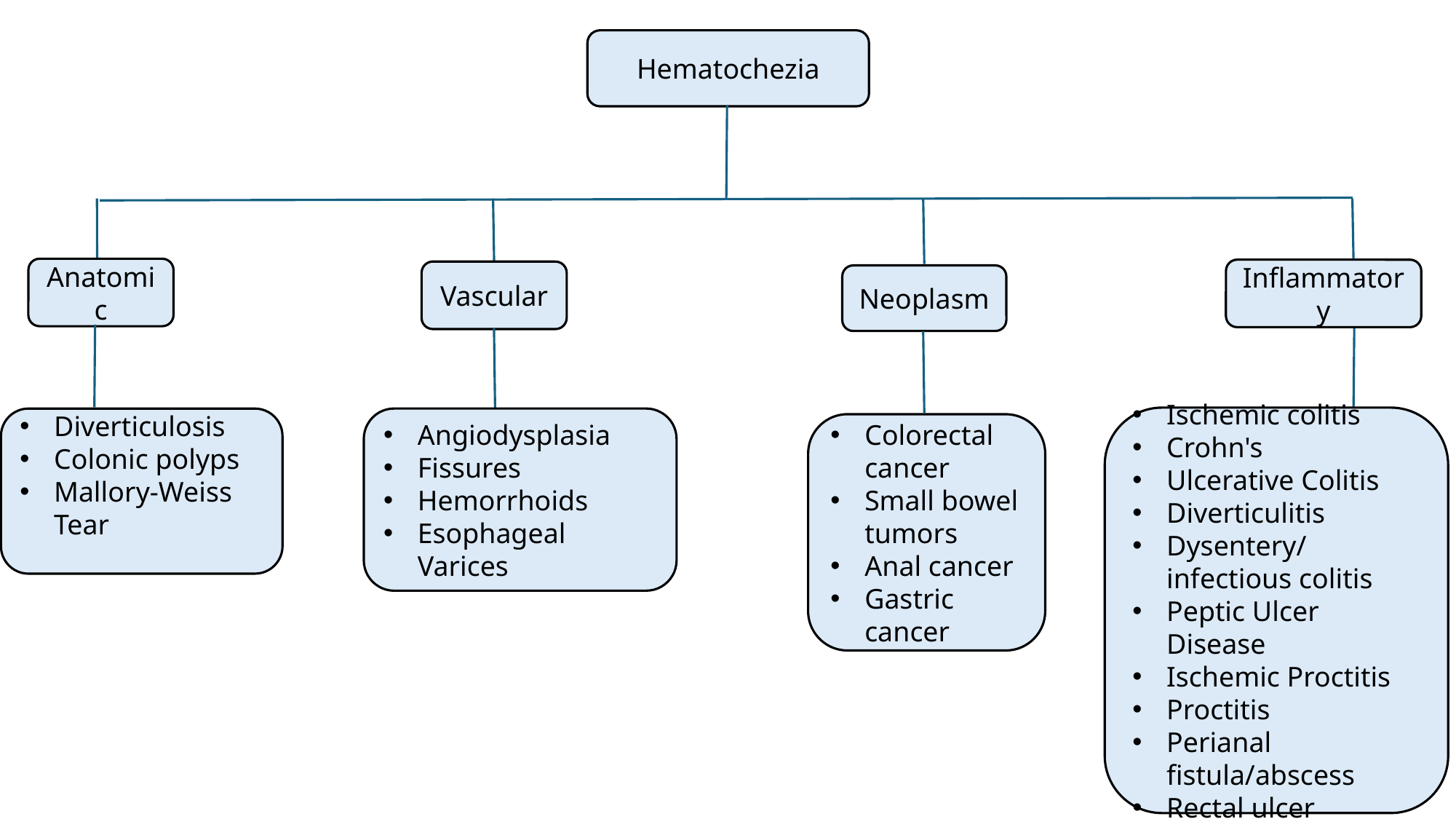

Hematochezia
Anatomic
Inflammatory
Vascular
Neoplasm
Ischemic colitis
Crohn's
Ulcerative Colitis
Diverticulitis
Dysentery/infectious colitis
Peptic Ulcer Disease
Ischemic Proctitis
Proctitis
Perianal fistula/abscess
Rectal ulcer
Diverticulosis
Colonic polyps
Mallory-Weiss Tear
Angiodysplasia
Fissures
Hemorrhoids
Esophageal Varices
Colorectal cancer
Small bowel tumors
Anal cancer
Gastric cancer

## Slide 16
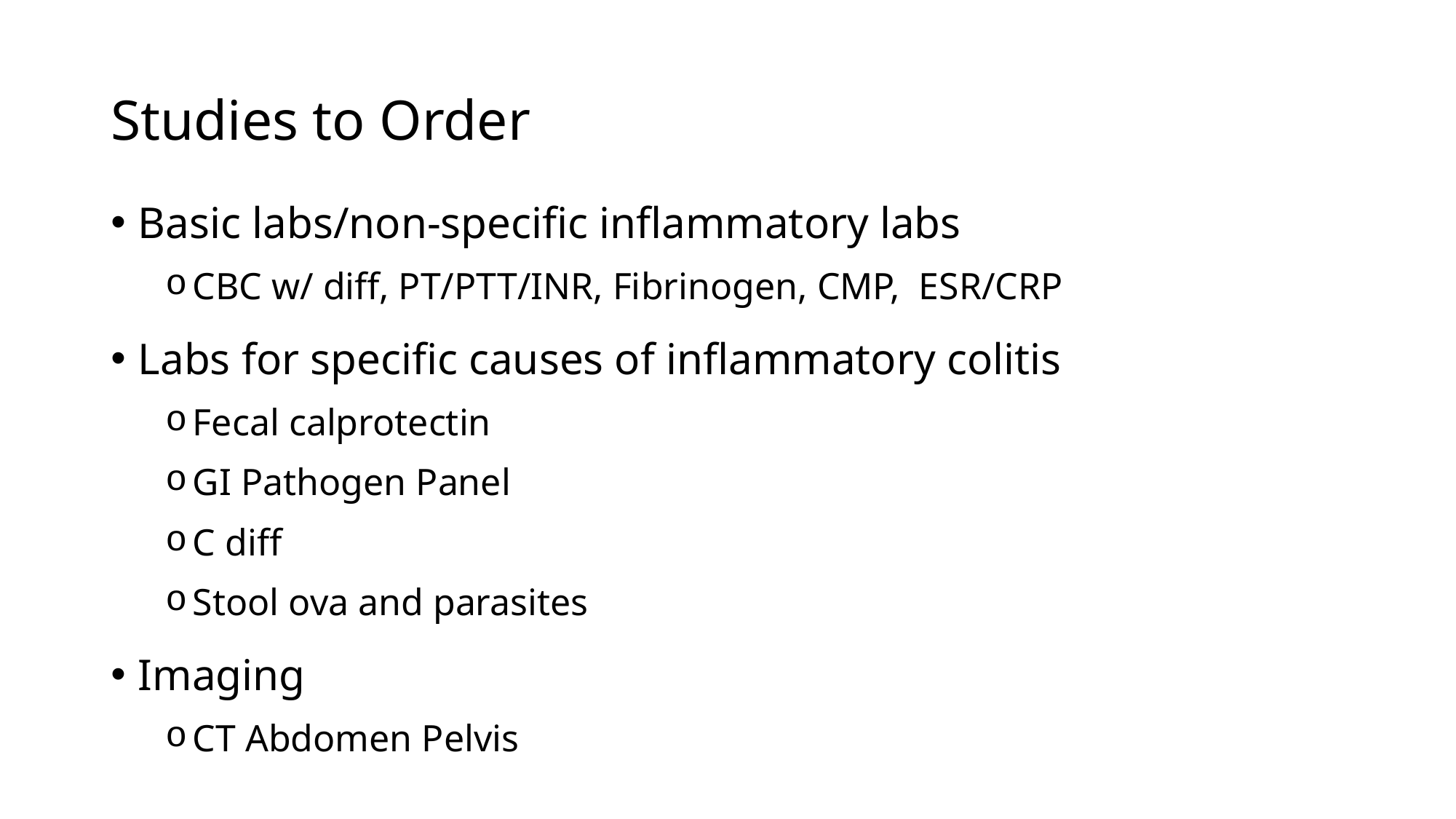

# Studies to Order
Basic labs/non-specific inflammatory labs
CBC w/ diff, PT/PTT/INR, Fibrinogen, CMP,  ESR/CRP
Labs for specific causes of inflammatory colitis
Fecal calprotectin
GI Pathogen Panel
C diff
Stool ova and parasites
Imaging
CT Abdomen Pelvis

## Slide 17
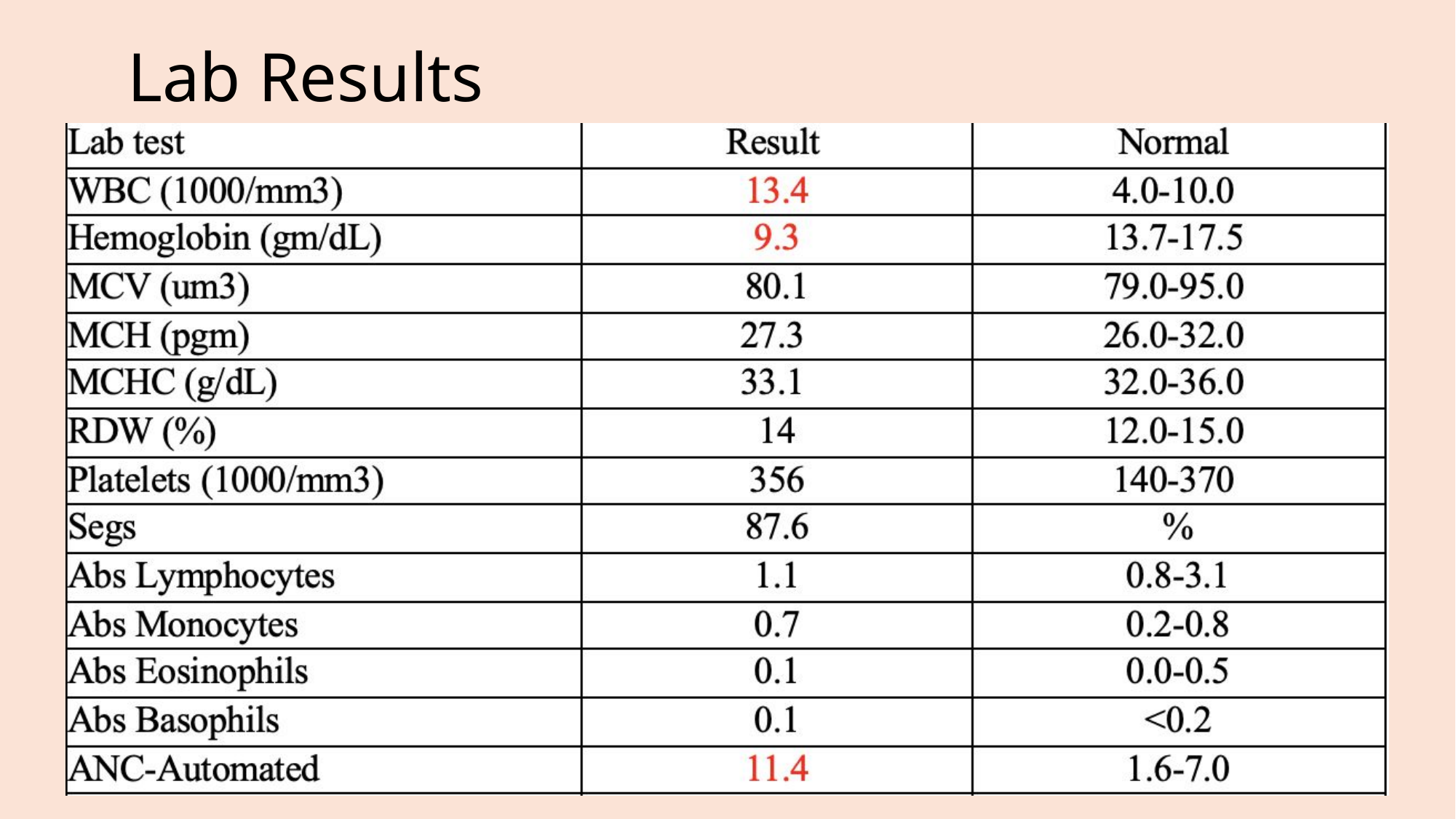

# Lab Results

## Slide 18
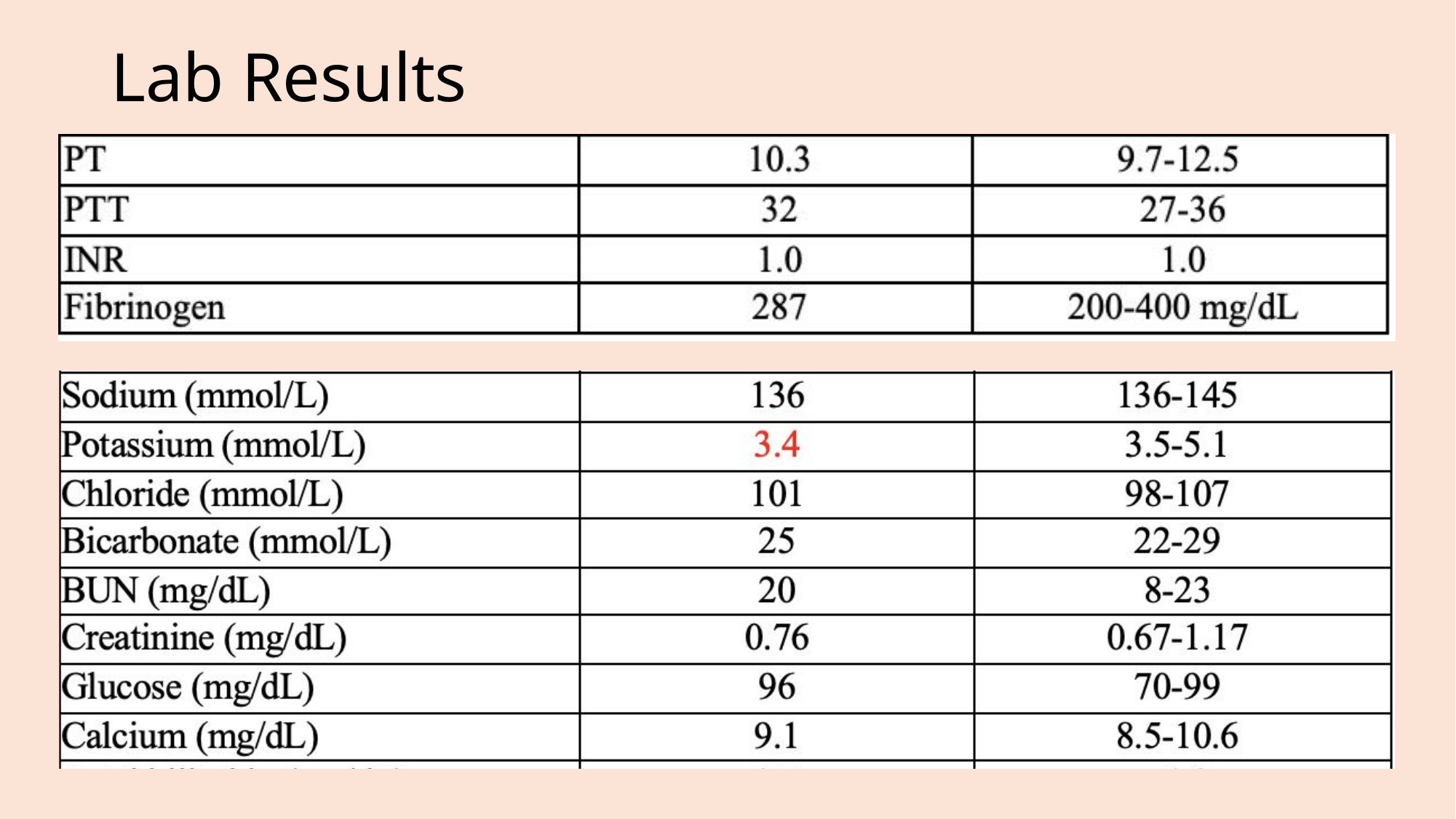

# Lab Results

## Slide 19
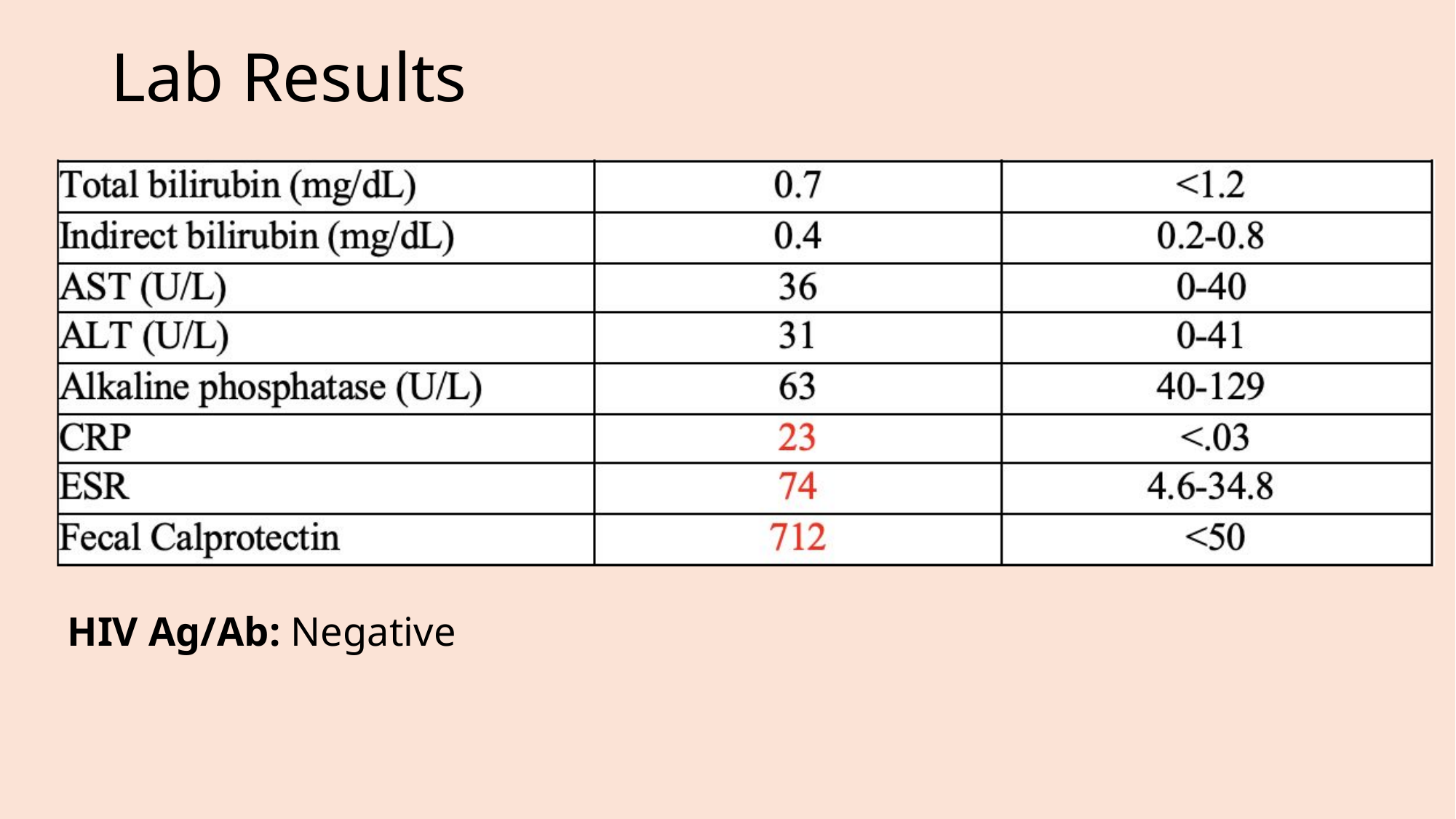

# Lab Results
HIV Ag/Ab: Negative

## Slide 20
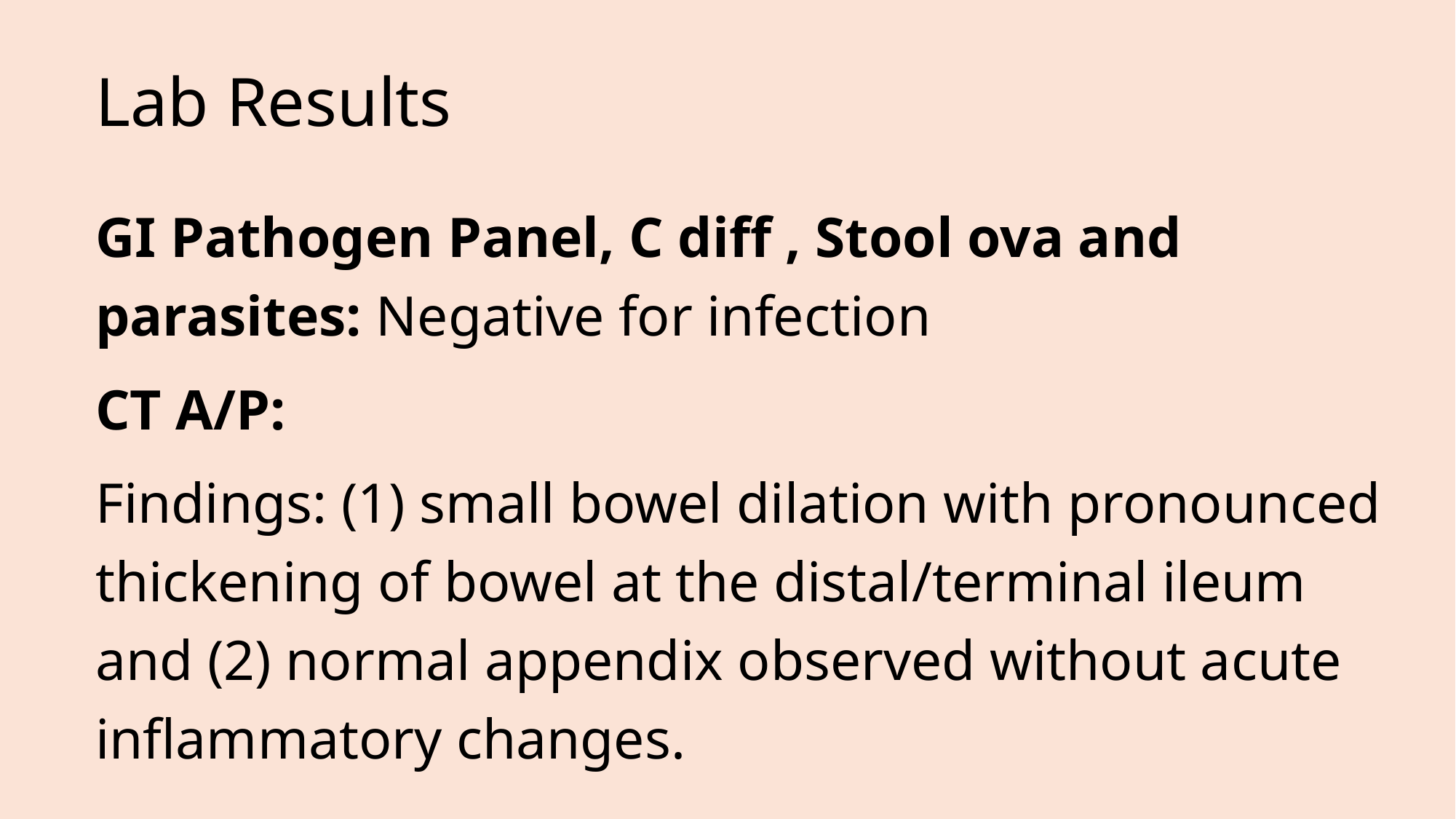

# Lab Results
GI Pathogen Panel, C diff , Stool ova and parasites: Negative for infection
CT A/P:
Findings: (1) small bowel dilation with pronounced thickening of bowel at the distal/terminal ileum and (2) normal appendix observed without acute inflammatory changes.

## Slide 21
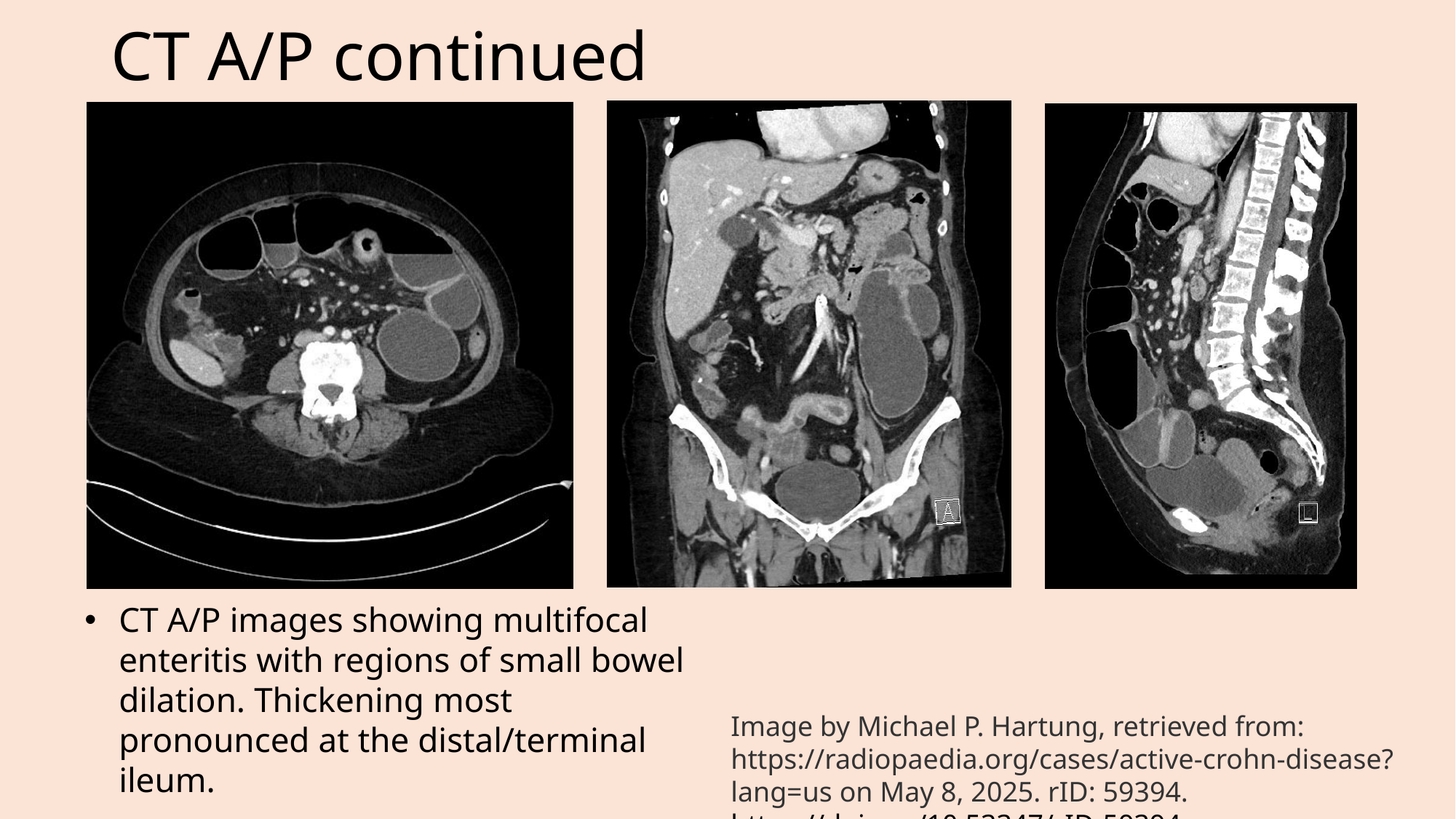

# CT A/P continued
CT A/P images showing multifocal enteritis with regions of small bowel dilation. Thickening most pronounced at the distal/terminal ileum.
Image by Michael P. Hartung, retrieved from: https://radiopaedia.org/cases/active-crohn-disease?lang=us on May 8, 2025. rID: 59394. https://doi.org/10.53347/rID-59394

## Slide 22
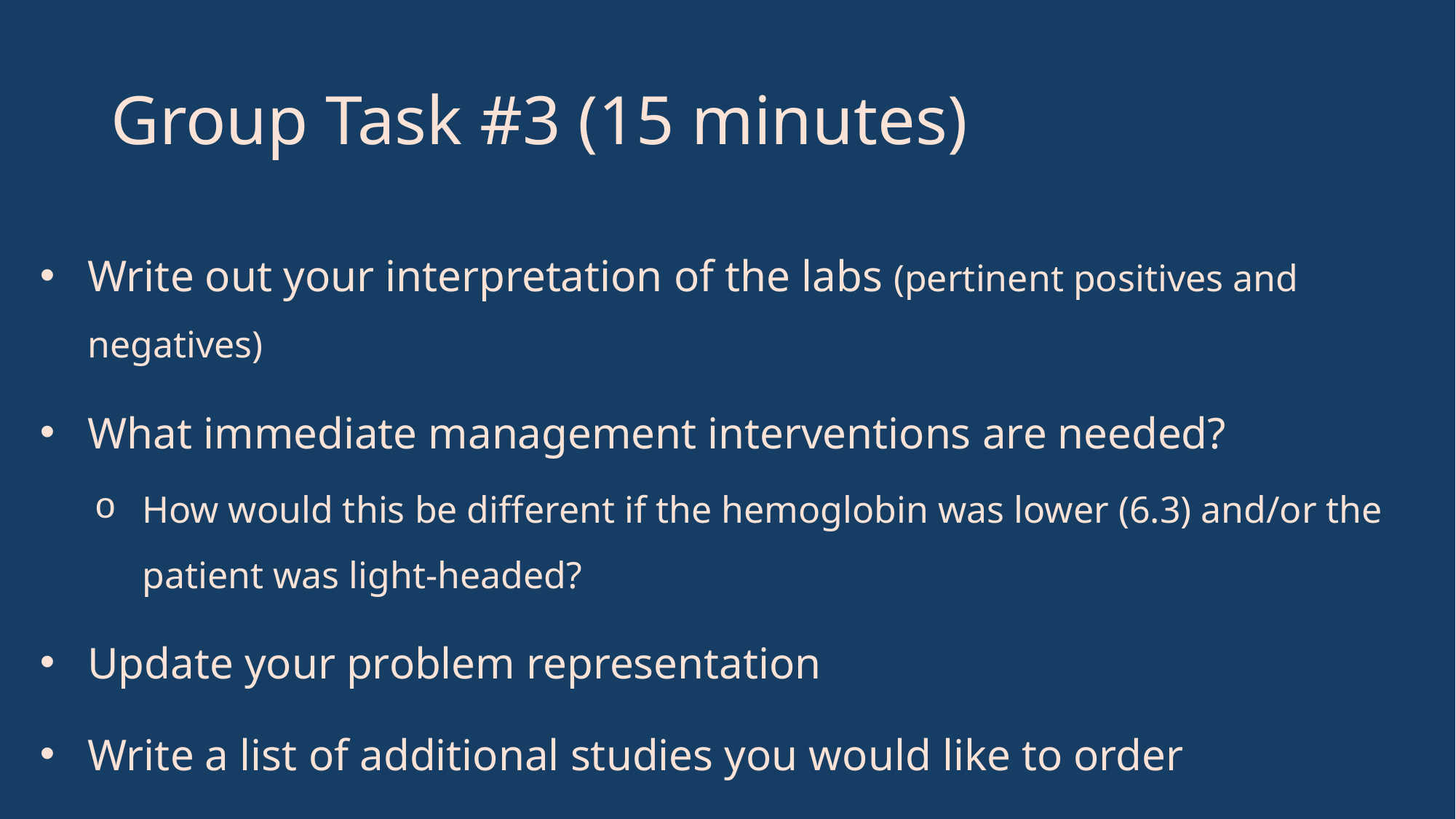

# Group Task #3 (15 minutes)
Write out your interpretation of the labs (pertinent positives and negatives)
What immediate management interventions are needed?
How would this be different if the hemoglobin was lower (6.3) and/or the patient was light-headed?
Update your problem representation
Write a list of additional studies you would like to order

## Slide 23
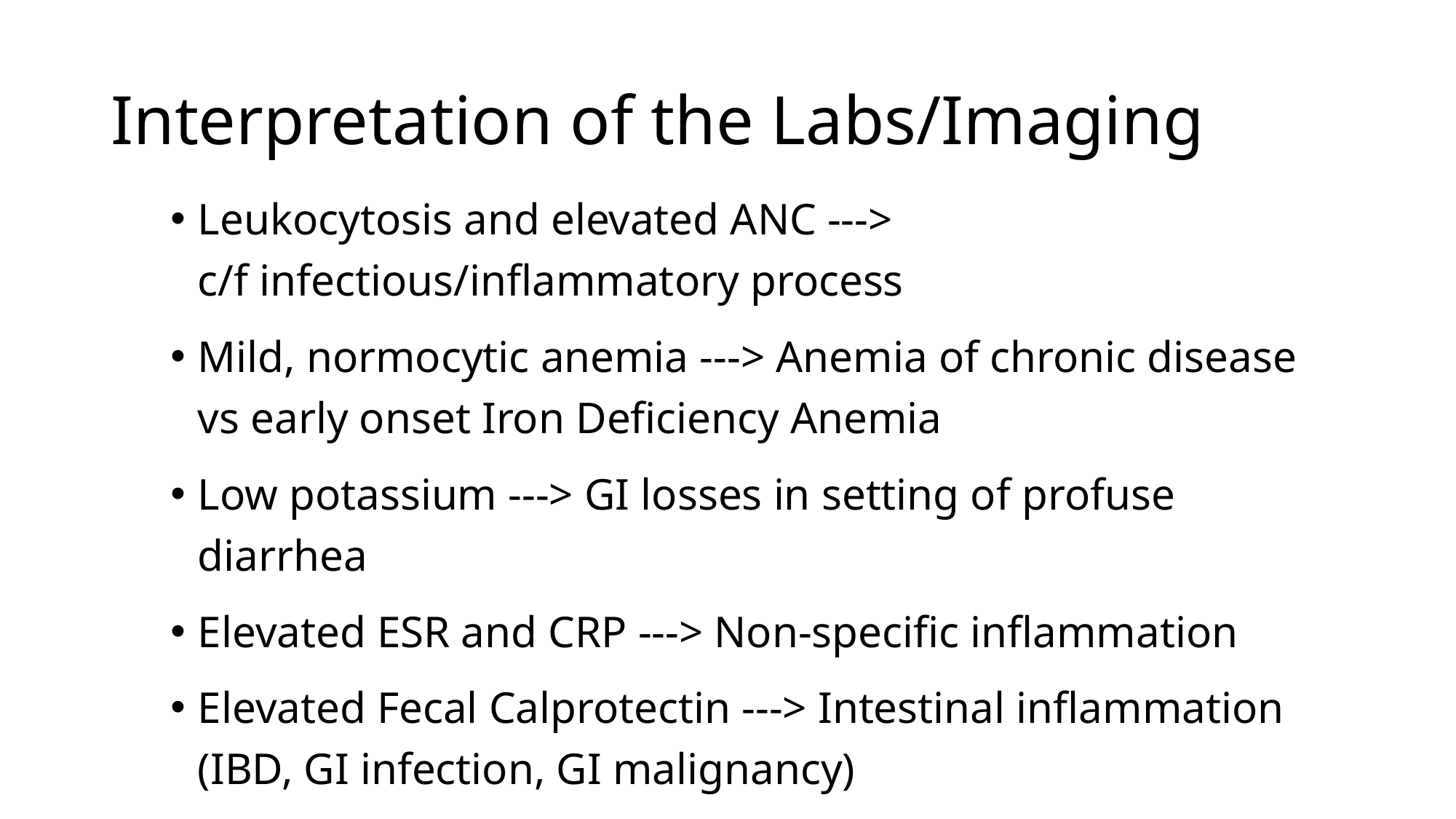

# Interpretation of the Labs/Imaging
Leukocytosis and elevated ANC ---> c/f infectious/inflammatory process
Mild, normocytic anemia ---> Anemia of chronic disease vs early onset Iron Deficiency Anemia
Low potassium ---> GI losses in setting of profuse diarrhea
Elevated ESR and CRP ---> Non-specific inflammation
Elevated Fecal Calprotectin ---> Intestinal inflammation (IBD, GI infection, GI malignancy)

## Slide 24
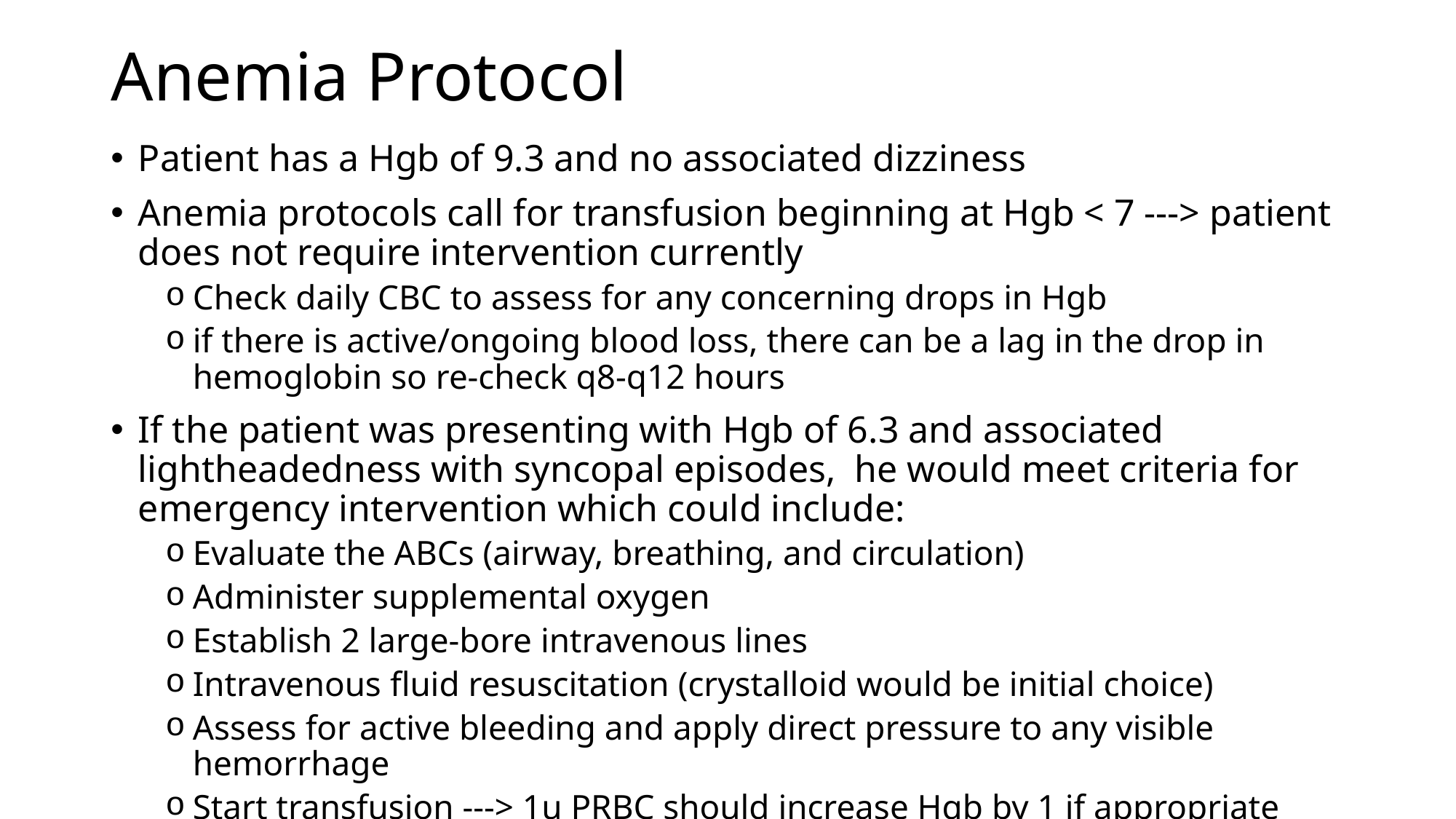

# Anemia Protocol
Patient has a Hgb of 9.3 and no associated dizziness
Anemia protocols call for transfusion beginning at Hgb < 7 ---> patient does not require intervention currently
Check daily CBC to assess for any concerning drops in Hgb
if there is active/ongoing blood loss, there can be a lag in the drop in hemoglobin so re-check q8-q12 hours
If the patient was presenting with Hgb of 6.3 and associated lightheadedness with syncopal episodes,  he would meet criteria for emergency intervention which could include:
Evaluate the ABCs (airway, breathing, and circulation)
Administer supplemental oxygen
Establish 2 large-bore intravenous lines
Intravenous fluid resuscitation (crystalloid would be initial choice)
Assess for active bleeding and apply direct pressure to any visible hemorrhage
Start transfusion ---> 1u PRBC should increase Hgb by 1 if appropriate response

## Slide 25
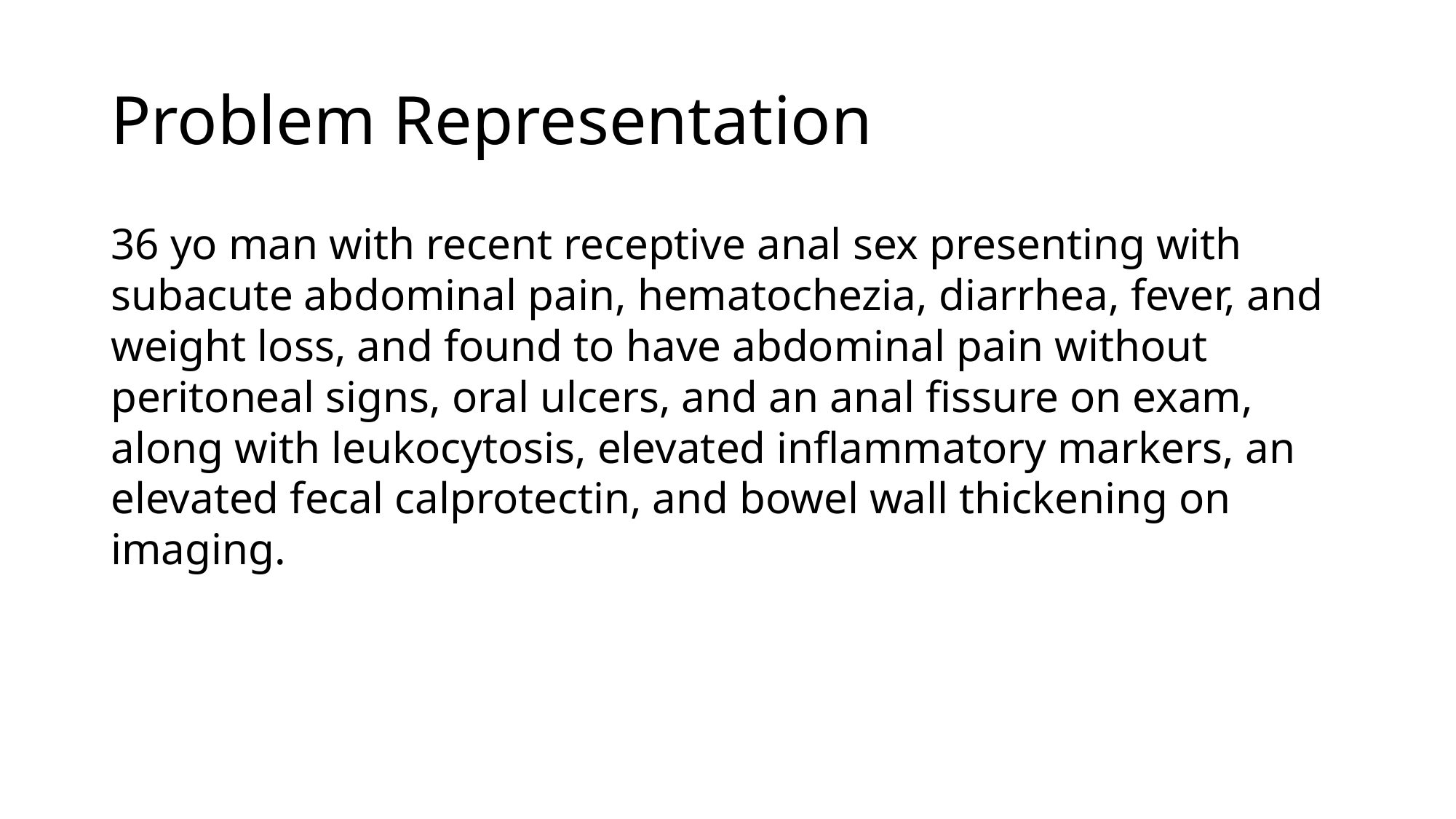

# Problem Representation
36 yo man with recent receptive anal sex presenting with subacute abdominal pain, hematochezia, diarrhea, fever, and weight loss, and found to have abdominal pain without peritoneal signs, oral ulcers, and an anal fissure on exam, along with leukocytosis, elevated inflammatory markers, an elevated fecal calprotectin, and bowel wall thickening on imaging.

## Slide 26
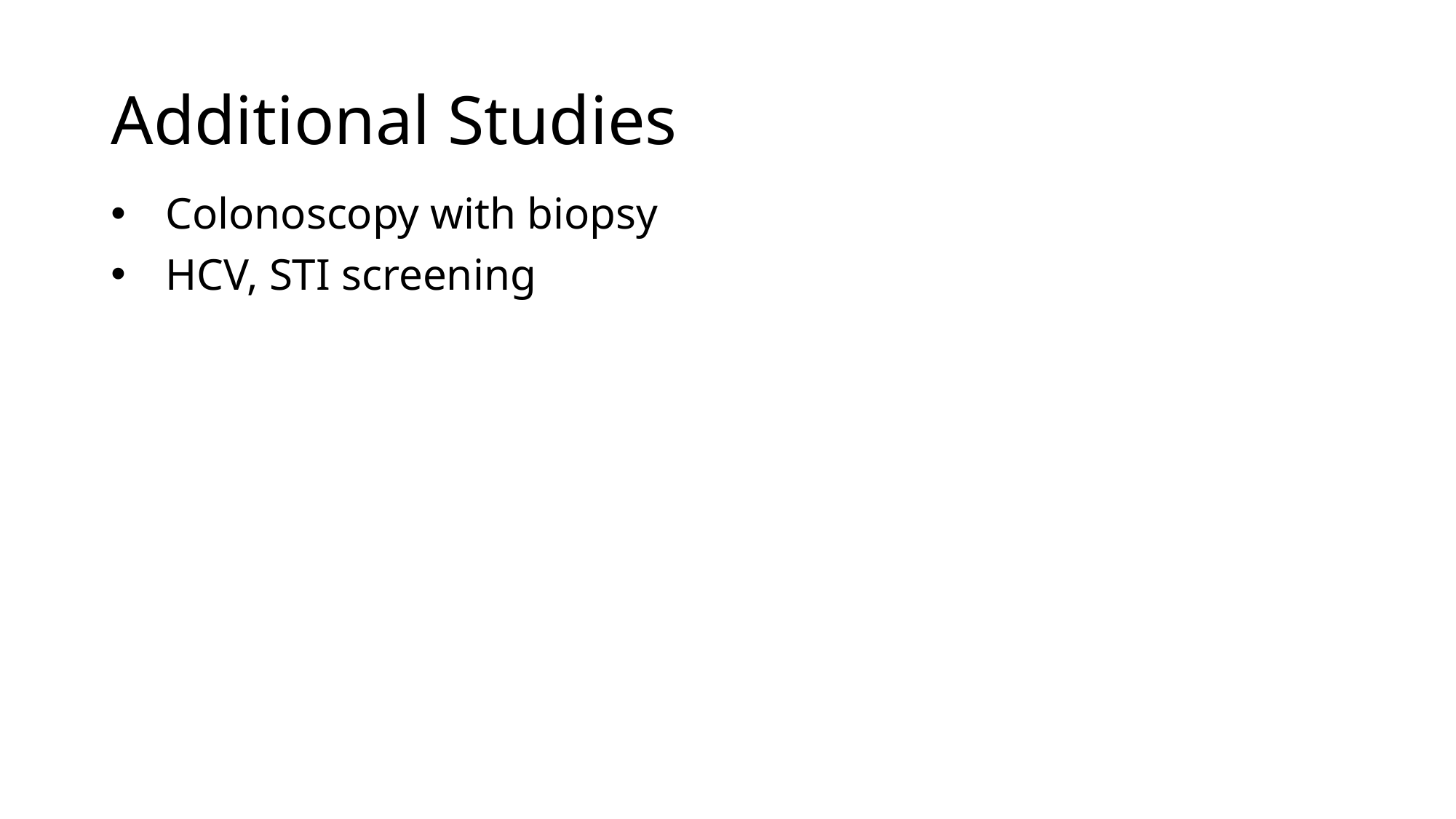

# Additional Studies
Colonoscopy with biopsy
HCV, STI screening

## Slide 27
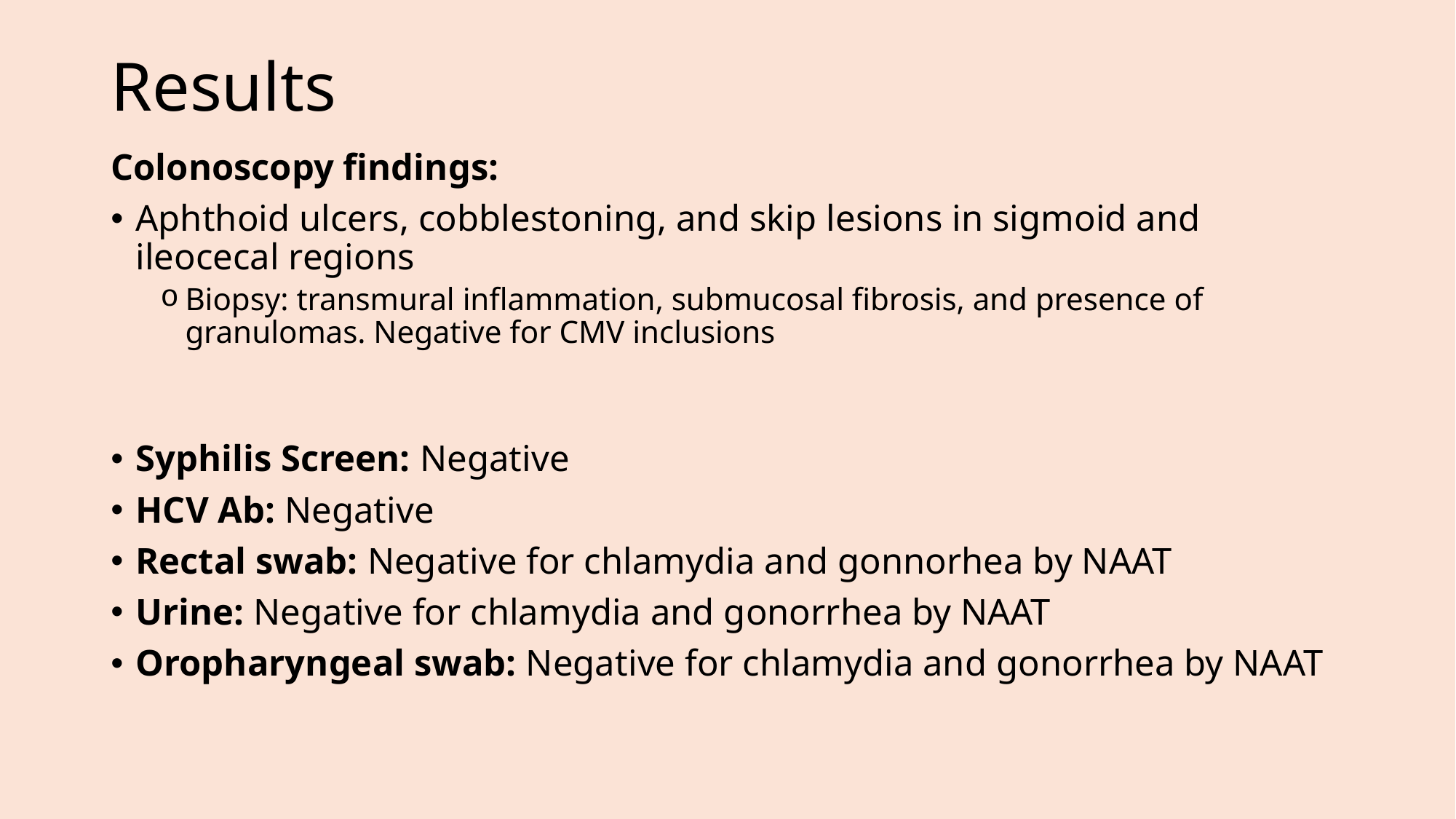

# Results
Colonoscopy findings:
Aphthoid ulcers, cobblestoning, and skip lesions in sigmoid and ileocecal regions
Biopsy: transmural inflammation, submucosal fibrosis, and presence of granulomas. Negative for CMV inclusions
Syphilis Screen: Negative
HCV Ab: Negative
Rectal swab: Negative for chlamydia and gonnorhea by NAAT
Urine: Negative for chlamydia and gonorrhea by NAAT
Oropharyngeal swab: Negative for chlamydia and gonorrhea by NAAT

## Slide 28
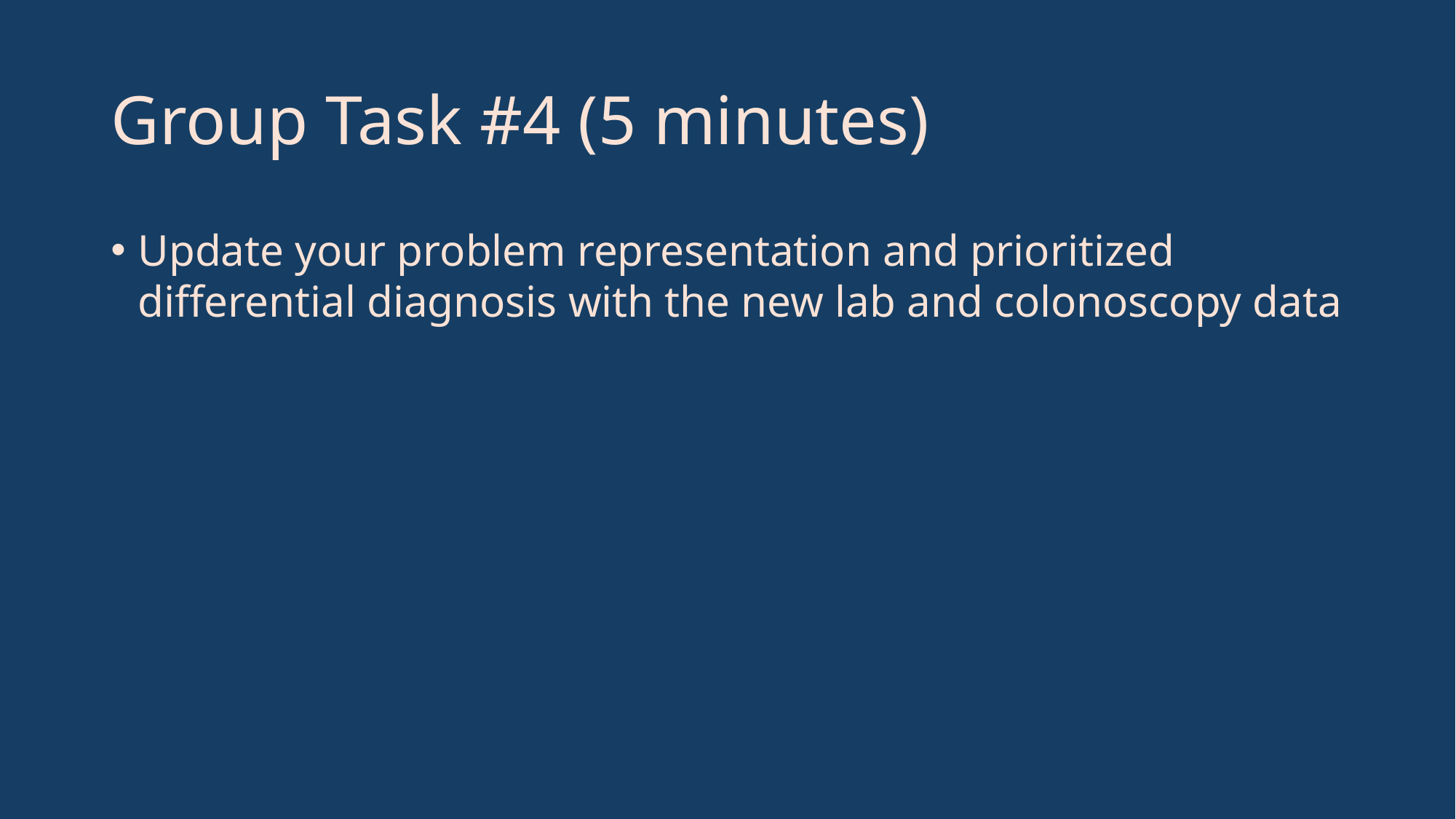

# Group Task #4 (5 minutes)
Update your problem representation and prioritized differential diagnosis with the new lab and colonoscopy data

## Slide 29
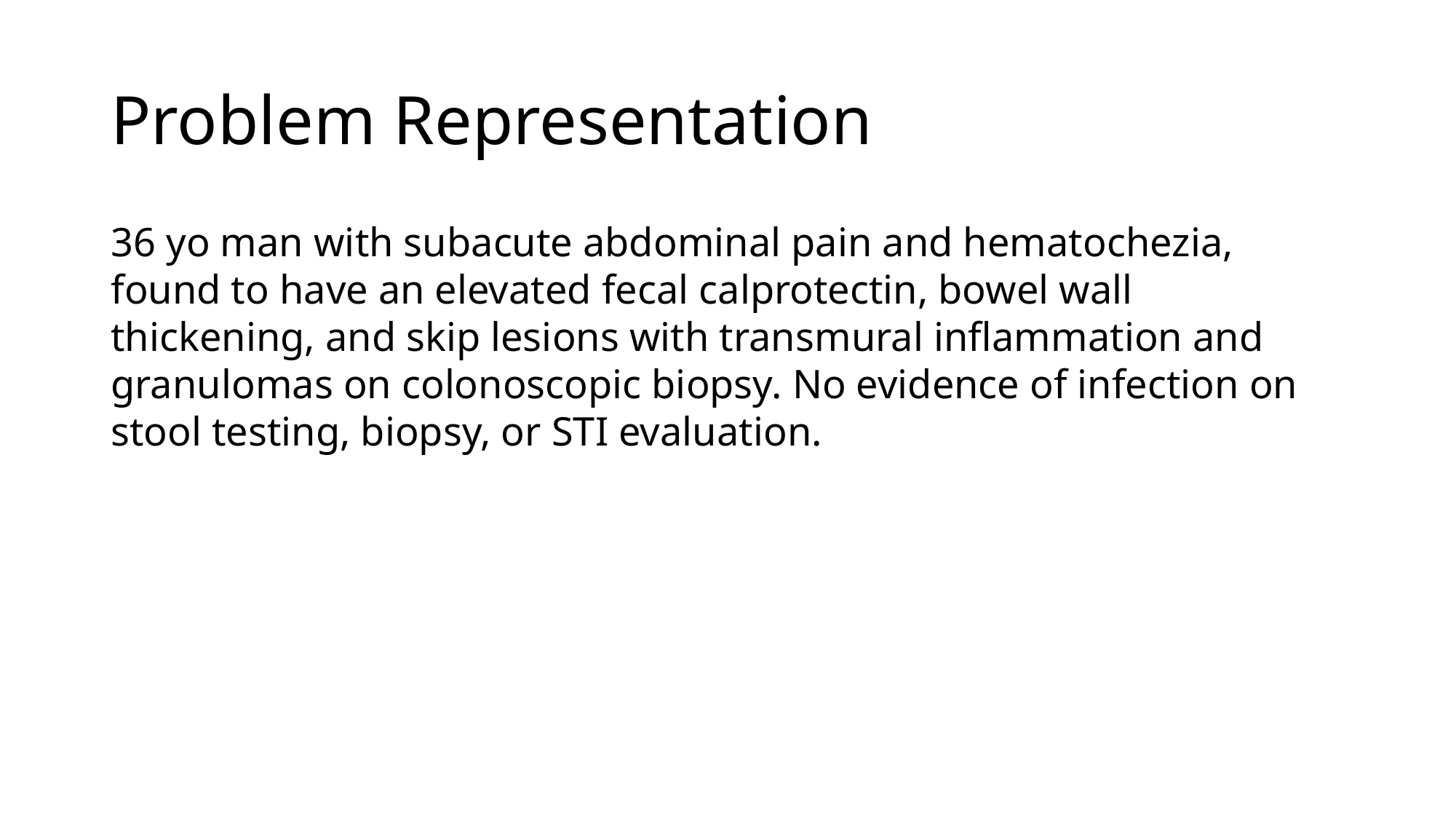

# Problem Representation
36 yo man with subacute abdominal pain and hematochezia, found to have an elevated fecal calprotectin, bowel wall thickening, and skip lesions with transmural inflammation and granulomas on colonoscopic biopsy. No evidence of infection on stool testing, biopsy, or STI evaluation.

## Slide 30
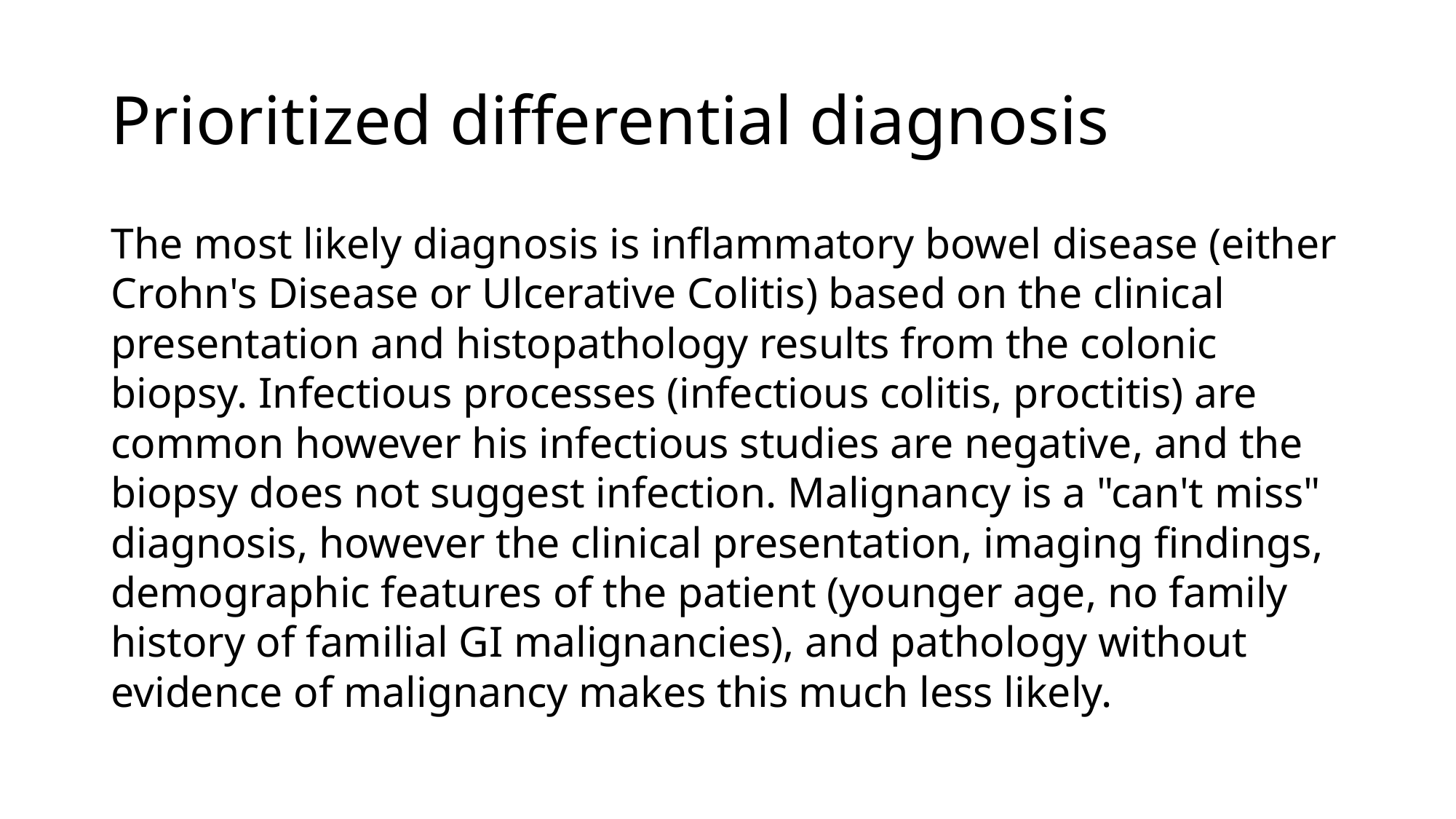

# Prioritized differential diagnosis
The most likely diagnosis is inflammatory bowel disease (either Crohn's Disease or Ulcerative Colitis) based on the clinical presentation and histopathology results from the colonic biopsy. Infectious processes (infectious colitis, proctitis) are common however his infectious studies are negative, and the biopsy does not suggest infection. Malignancy is a "can't miss" diagnosis, however the clinical presentation, imaging findings, demographic features of the patient (younger age, no family history of familial GI malignancies), and pathology without evidence of malignancy makes this much less likely.

## Slide 31
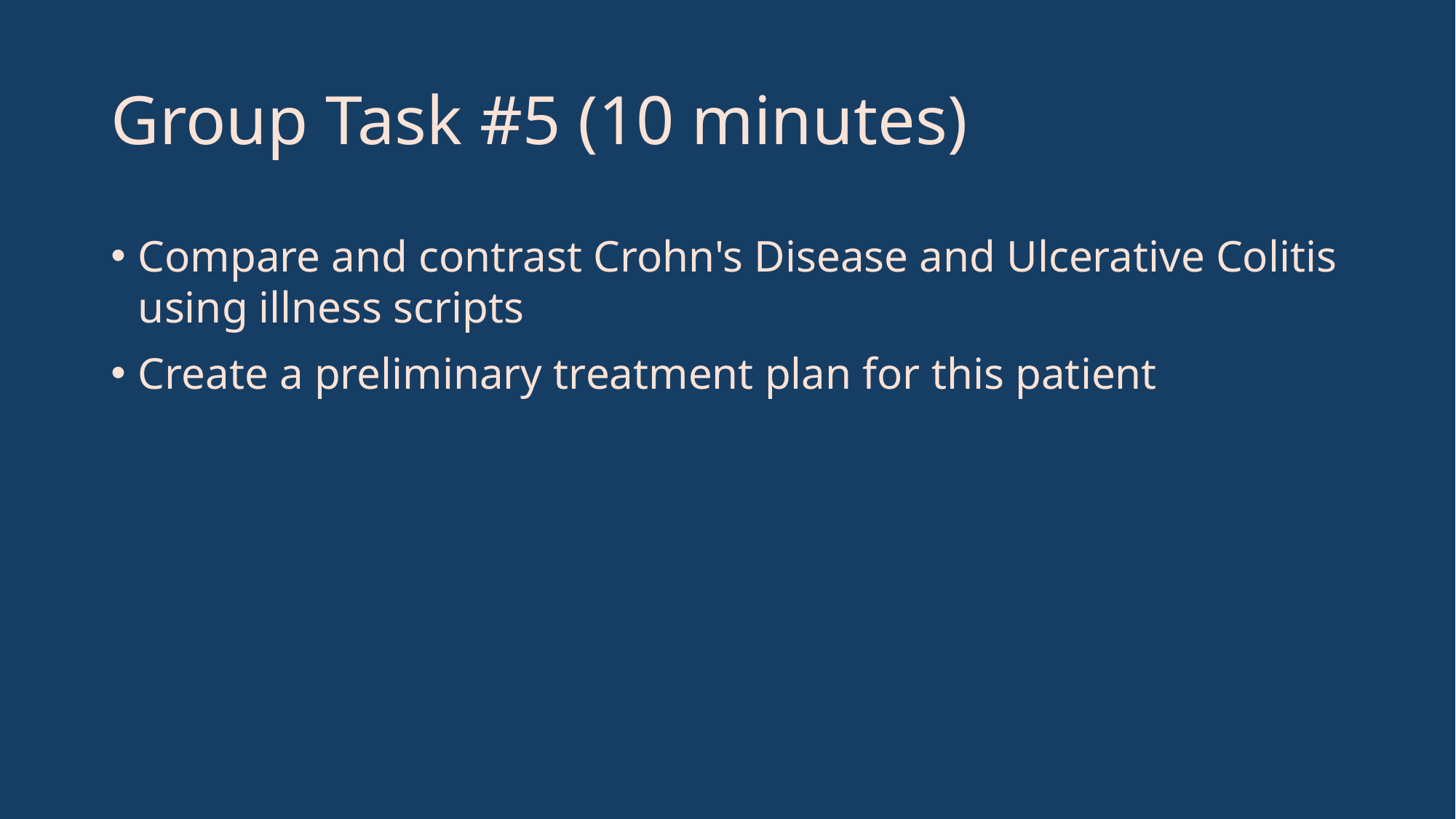

# Group Task #5 (10 minutes)
Compare and contrast Crohn's Disease and Ulcerative Colitis using illness scripts
Create a preliminary treatment plan for this patient

## Slide 32
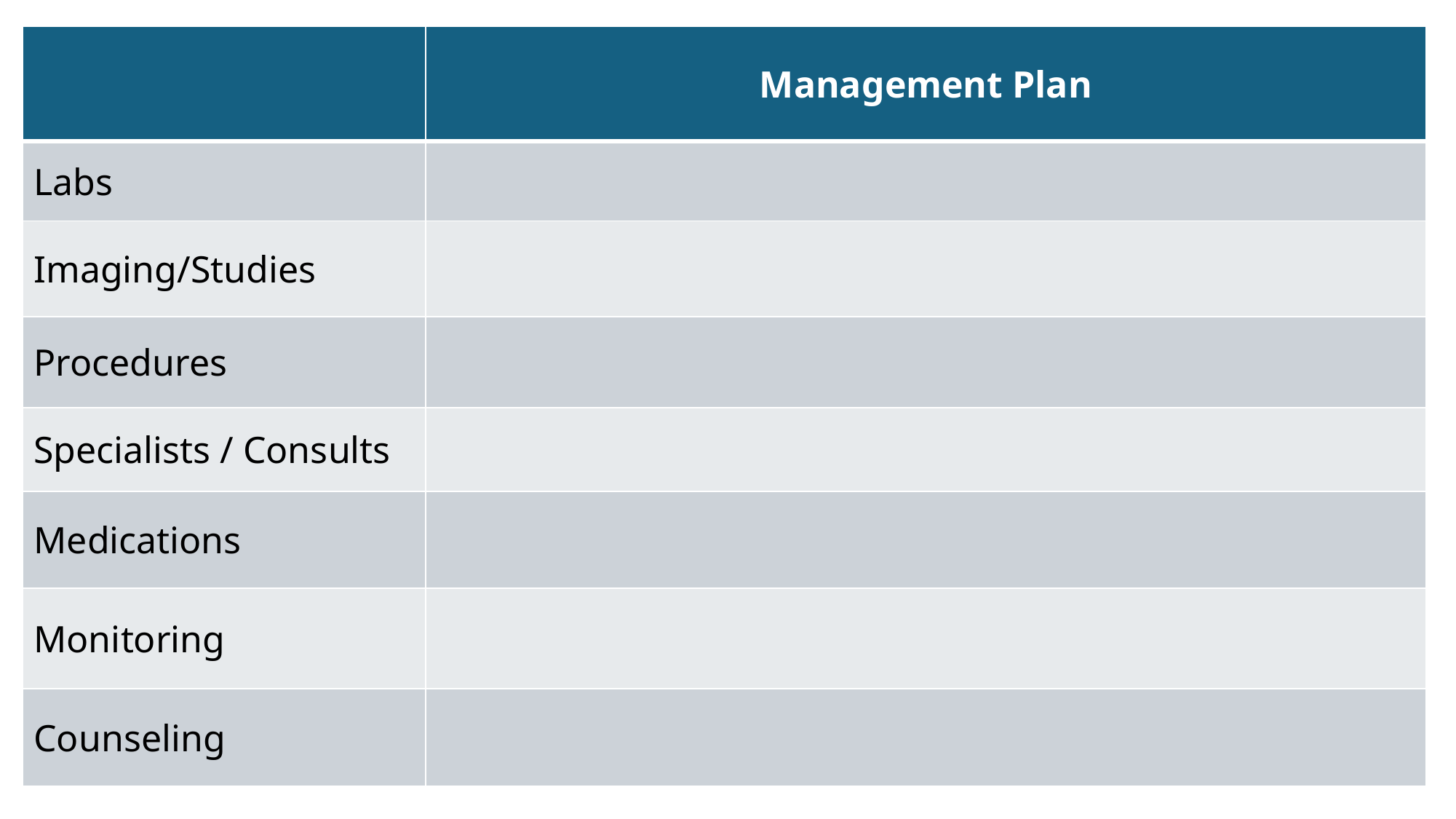

| | Management Plan |
| --- | --- |
| Labs | |
| Imaging/Studies | |
| Procedures | |
| Specialists / Consults | |
| Medications | |
| Monitoring | |
| Counseling | |

## Slide 33
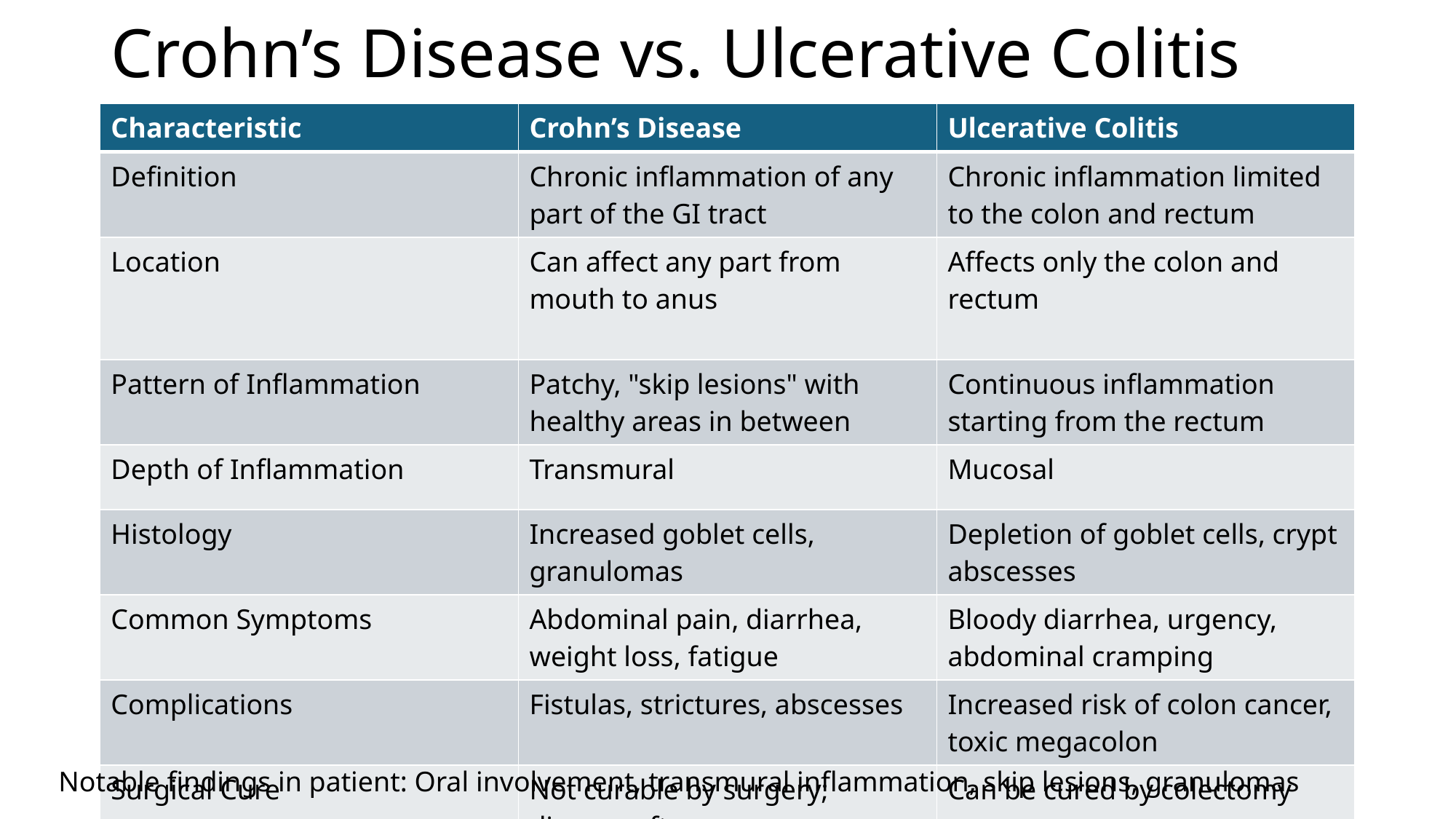

# Crohn’s Disease vs. Ulcerative Colitis
| Characteristic | Crohn’s Disease | Ulcerative Colitis |
| --- | --- | --- |
| Definition | Chronic inflammation of any part of the GI tract | Chronic inflammation limited to the colon and rectum |
| Location | Can affect any part from mouth to anus | Affects only the colon and rectum |
| Pattern of Inflammation | Patchy, "skip lesions" with healthy areas in between | Continuous inflammation starting from the rectum |
| Depth of Inflammation | Transmural | Mucosal |
| Histology | Increased goblet cells, granulomas | Depletion of goblet cells, crypt abscesses |
| Common Symptoms | Abdominal pain, diarrhea, weight loss, fatigue | Bloody diarrhea, urgency, abdominal cramping |
| Complications | Fistulas, strictures, abscesses | Increased risk of colon cancer, toxic megacolon |
| Surgical Cure | Not curable by surgery; disease often recurs | Can be cured by colectomy |
Notable findings in patient: Oral involvement, transmural inflammation, skip lesions, granulomas

## Slide 34
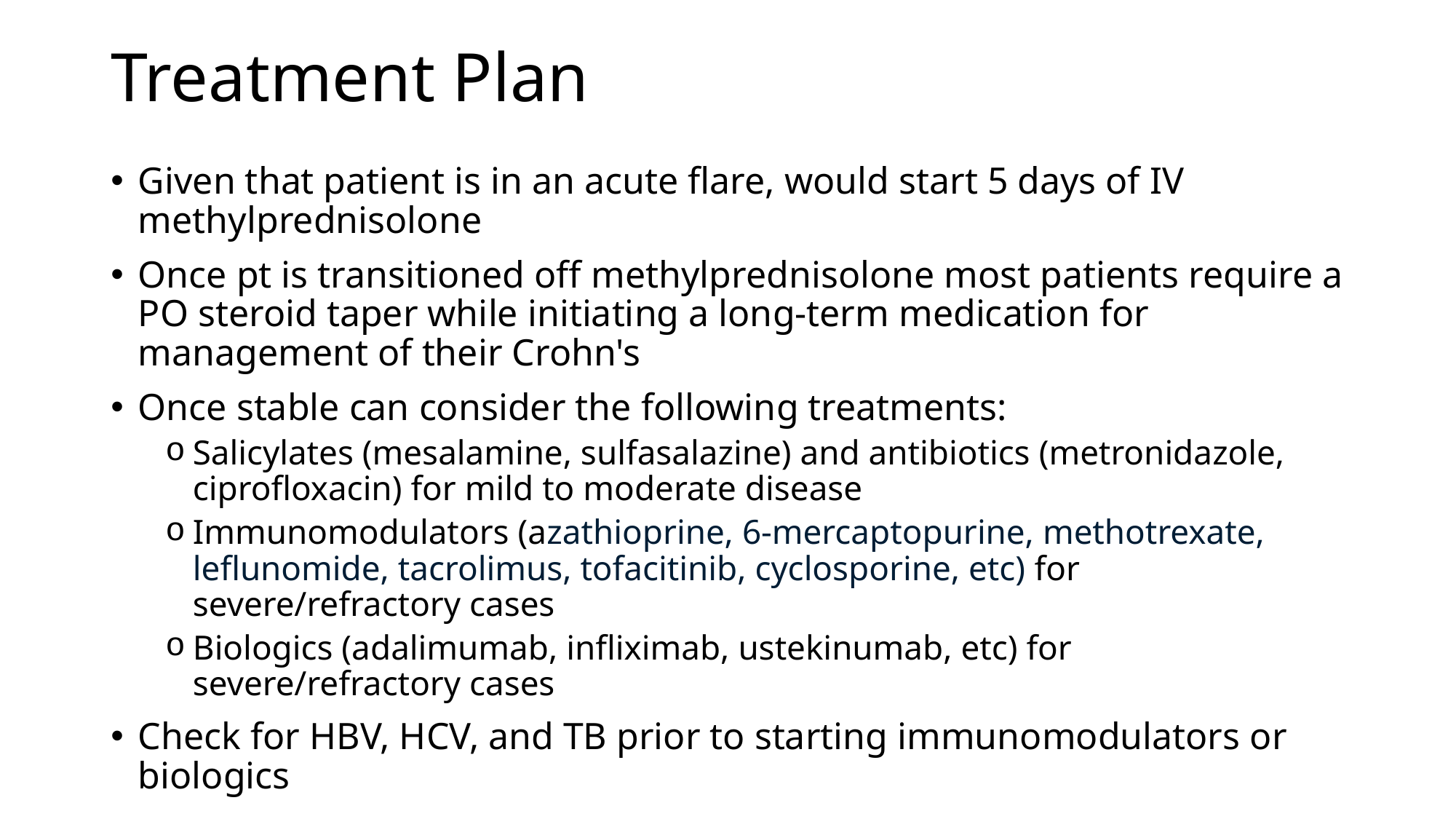

# Treatment Plan
Given that patient is in an acute flare, would start 5 days of IV methylprednisolone
Once pt is transitioned off methylprednisolone most patients require a PO steroid taper while initiating a long-term medication for management of their Crohn's
Once stable can consider the following treatments:
Salicylates (mesalamine, sulfasalazine) and antibiotics (metronidazole, ciprofloxacin) for mild to moderate disease
Immunomodulators (azathioprine, 6-mercaptopurine, methotrexate, leflunomide, tacrolimus, tofacitinib, cyclosporine, etc) for severe/refractory cases
Biologics (adalimumab, infliximab, ustekinumab, etc) for severe/refractory cases
Check for HBV, HCV, and TB prior to starting immunomodulators or biologics

## Slide 35
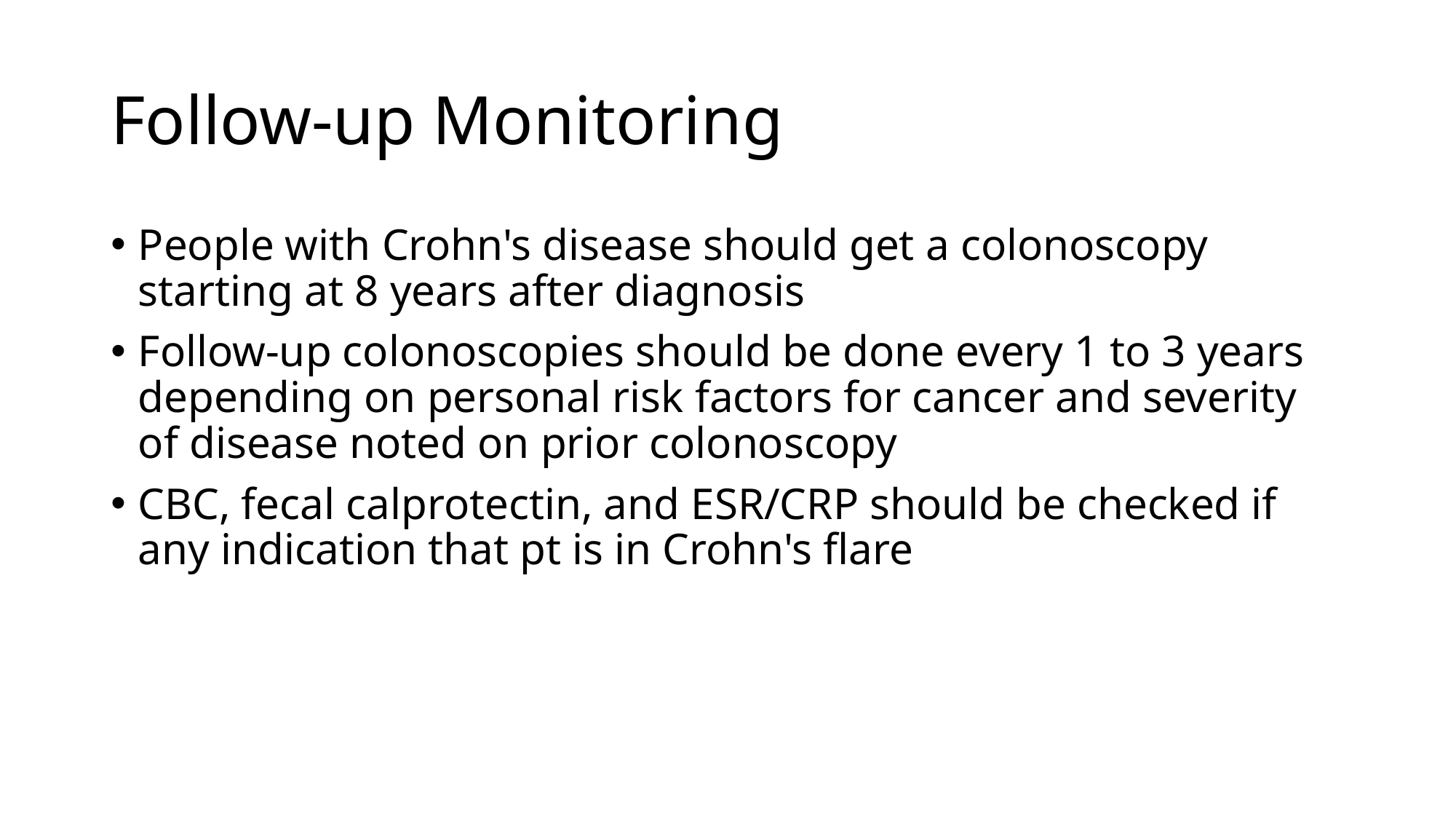

# Follow-up Monitoring
People with Crohn's disease should get a colonoscopy starting at 8 years after diagnosis
Follow-up colonoscopies should be done every 1 to 3 years depending on personal risk factors for cancer and severity of disease noted on prior colonoscopy
CBC, fecal calprotectin, and ESR/CRP should be checked if any indication that pt is in Crohn's flare

## Slide 36
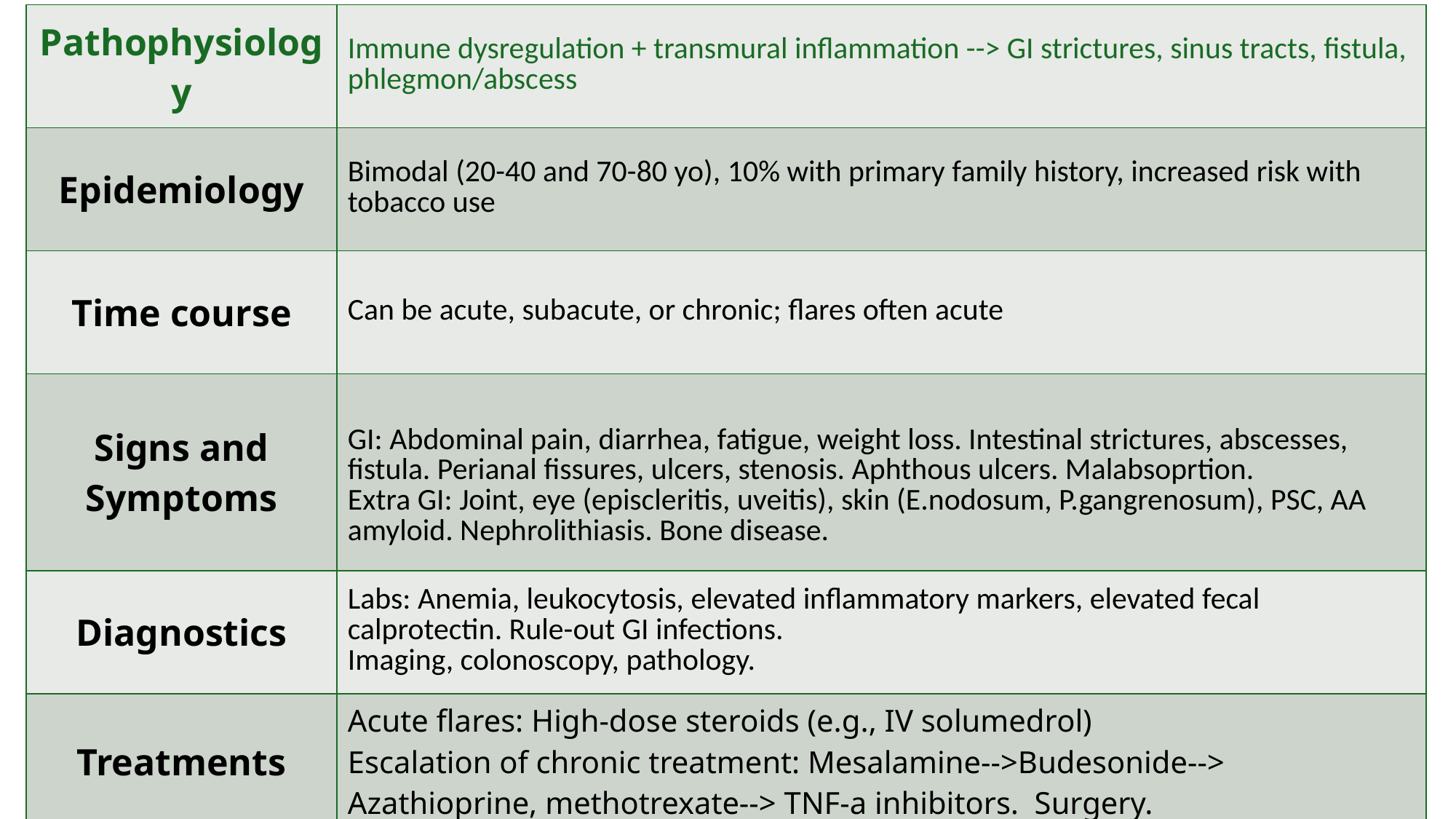

| Pathophysiology | Immune dysregulation + transmural inflammation --> GI strictures, sinus tracts, fistula, phlegmon/abscess |
| --- | --- |
| Epidemiology | Bimodal (20-40 and 70-80 yo), 10% with primary family history, increased risk with tobacco use |
| Time course | Can be acute, subacute, or chronic; flares often acute |
| Signs and Symptoms | GI: Abdominal pain, diarrhea, fatigue, weight loss. Intestinal strictures, abscesses, fistula. Perianal fissures, ulcers, stenosis. Aphthous ulcers. Malabsoprtion. Extra GI: Joint, eye (episcleritis, uveitis), skin (E.nodosum, P.gangrenosum), PSC, AA amyloid. Nephrolithiasis. Bone disease. |
| Diagnostics | Labs: Anemia, leukocytosis, elevated inflammatory markers, elevated fecal calprotectin. Rule-out GI infections. Imaging, colonoscopy, pathology. |
| Treatments | Acute flares: High-dose steroids (e.g., IV solumedrol) Escalation of chronic treatment: Mesalamine-->Budesonide--> Azathioprine, methotrexate--> TNF-a inhibitors. Surgery. |
